# Small-Scale Heterogeneity in Drinking Water Biofilms

**DOI:** 10.3389/fmicb.2019.02446

**Published:** 2019-10-29

**Authors:** Lisa Neu, Caitlin R. Proctor, Jean-Claude Walser, Frederik Hammes

**Affiliations:** ^1^Department of Environmental Microbiology, Eawag: Swiss Federal Institute of Aquatic Science and Technology, Dübendorf, Switzerland; ^2^Department of Environmental Systems Science, Institute of Biogeochemistry and Pollutant Dynamics, ETH Zürich, Zurich, Switzerland; ^3^Schools of Civil, Environmental and Ecological, Materials, and Biomedical Engineering, Purdue University, West Lafayette, IN, United States; ^4^Genetic Diversity Centre (GDC), ETH Zürich, Zurich, Switzerland

**Keywords:** drinking water, biofilm, small-scale heterogeneity, microbiome, selection

## Abstract

Biofilm heterogeneity has been characterized on various scales for both natural and engineered ecosystems. This heterogeneity has been attributed to spatial differences in environmental factors. Understanding their impact on localized biofilm heterogeneity in building plumbing systems is important for both management and representative sampling strategies. We assessed heterogeneity within the confined engineered ecosystem of a shower hose by high-resolution sampling (200 individual biofilm sections per hose) on varying scales (μm to m). We postulated that a biofilm grown on a single material under uniform conditions should be homogeneous in its structure, bacterial numbers, and community composition. A biofilm grown for 12 months under controlled laboratory conditions, showed homogeneity on large-scale. However, some small-scale heterogeneity was clearly observed. For example, biofilm thickness of cm-sections varied up to 4-fold, total cell concentrations (TCC) 3-fold, and relative abundance of dominant taxa up to 5-fold. A biofilm grown under real (i.e., uncontrolled) use conditions developed considerably more heterogeneity in all variables which was attributed to more discontinuity in environmental conditions. Interestingly, biofilm communities from both hoses showed comparably low diversity, with <400 taxa each, and only three taxa accounting for 57%, respectively, 73% of the community. This low diversity was attributed to a strong selective pressure, originating in migrating carbon from the flexible hoses as major carbon source. High-resolution sampling strategy enabled detailed analysis of spatial heterogeneity within an individual drinking water biofilm. This study gives insight into biofilm structure and community composition on cm-to m-scale and is useful for decision-making on sampling strategies in biofilm research and monitoring.

## Introduction

Microbial biogeography has been documented in diverse aquatic ecosystems and on various spatial scales (Roeselers et al., 2015; Liu et al., 2018). Numerous studies revealed a remarkable heterogeneity (i.e., variations) in bacterial abundance (Liu et al., 2013; Siles and Margesin, 2016), metabolic activities (Chao et al., 2015; Charlop-Powers et al., 2015), or microbiomes (Stanish et al., 2016; Boers et al., 2018). Interestingly, this heterogeneity was not attributed to distance *per se*, but mainly to spatial differences in environmental factors (Hou et al., 2017; Langenheder et al., 2017). For example, biogeographical heterogeneity in natural freshwater ecosystems was shown to be driven by localized differences in factors such as temperature (Liu et al., 2018), alkalinity (Langenheder et al., 2017), and salinity (Berga et al., 2017).

Many environmental factors that enable biogeographical heterogeneity in natural ecosystems are equally relevant in confined engineered aquatic ecosystems, such as drinking water treatment and distribution systems. For example, heterogeneity was ascribed to differences in treatment processes, e.g., treatment units (Ma et al., 2017; Bruno et al., 2018), filtration type (Pinto et al., 2012; Lautenschlager et al., 2014), or filtration media (Vignola et al., 2018). Also, changes in the exposure to disinfection and disinfectant residuals (Servais et al., 2004; Potgieter et al., 2018), as well as differences in the composition and quantity of nutrients (Niquette et al., 2001; Bester et al., 2010), radial-spatial orientation (Lin et al., 2016; Liu et al., 2017), and temperature (Liu et al., 2013) were shown to cause biogeographical heterogeneity. The most dramatic variations in drinking water systems occur in the built environment. Here, several factors shape heterogeneous biofilms within the same connected system, namely: (1) diverse materials that support microbial growth (Liu R. et al., 2014; Wang et al., 2014) and select for material specific community compositions (Jang et al., 2011; Proctor et al., 2016), (2) variation in surface-to-volume ratios that increase microbial attachment/detachment rates/probabilities (Ling et al., 2018), (3) differences in flow/stagnation regimes (Lautenschlager et al., 2010; Douterelo et al., 2013), and (4) differences in water temperatures (Ji et al., 2017). These variations do not only occur between different sections of a system, but also within, e.g., one individual pipe or fixture. Considering the clear impact of variable environmental conditions on microbiology, it is reasonable to expect biogeographical heterogeneity within such a connected aquatic system. It is, however, less clear to what degree biogeographical heterogeneity can be expected when environmental factors are consistent, for example when a single pipe material is exposed to seemingly uniform environmental conditions along its whole length.

The goal of this study was to characterize spatial heterogeneity within a mature drinking water biofilm that grew inside a flexible shower hose. We aimed to identify environmental factors that shape biofilm heterogeneity and elucidate the importance of sample scale in both fundamental and applied biofilm research. Our hypothesis was that a biofilm grown on a single material under uniform conditions would be homogeneously distributed with respect to structure and composition. To test this, a biofilm was grown inside a flexible plastic hose (PVC-P) under defined and controlled laboratory conditions. Small-scale heterogeneity was assessed by comparing (1) biofilm structure and thickness, (2) total cell concentrations, and (3) bacterial community composition of a total of 200 sections of 1.2 cm. Additionally, a biofilm grown in an identical hose under real-use conditions was analyzed in the same way to assess the impact of more variable environmental conditions on biofilm spatial heterogeneity. Our sampling design enabled a high-resolution assessment of drinking water biofilms on small-scale, and the combination of quantitative and qualitative tools for biofilm characterization. This study provides a deeper insight into biofilm formation on building plumbing materials and consequently informs on biofilm sampling strategies.

## Materials and Methods

### Growing Biofilms Inside Flexible Shower Hoses Under Controlled and Real-Use Conditions

Biofilms were grown inside commercially available flexible shower hoses, purchased from the same batch of production. The hoses were made from plasticized polyvinyl chloride (PVC-P), with an inner diameter of 0.8 cm, a total length of 1.80 m, and originally with a metal cover outer sheath.

#### Biofilm Growth Under Controlled Laboratory Conditions

In the laboratory setup, the metal sheath was removed and the hose was horizontally aligned in a dark container, preventing any motion or physical disruption (further referred to as “control hose”). The installation was connected to a warm water tap with automated flushing events realized by a time-controlled magnetic valve. Over the course of one year, the hose was automatically flushed for 15 min with warm water (35–42°C) twice per day with consistent stagnation times of 8 and 16 h, respectively (Supplementary Figure S1). A flow velocity of 0.3 L/min was provided. The tap water was non-chlorinated drinking water, consisting mostly of pre-treated surface water (78.6%, Lake Zurich, ozonation, slow sand, activated carbon, and rapid sand filtration) and untreated groundwater (15%), with a small portion of pre-treated spring water (6%, UV disinfection) (Supplementary Table S1A).

#### Biofilm Growth Under Uncontrolled Real-Use Conditions

Complementary to the control hose, an identical PVC-P hose was installed in a real shower (further referred to as “real hose”), with the aim to assess the impact of more variable environmental conditions on biofilm heterogeneity. Usage habits (e.g., shower durations, stagnation times, water temperature, and flow rate) varied over the course of one year, with three residents sharing the shower. For showering, mixtures of warm and cold water lines were used with varying and higher flow velocities compared to the control hose (average use: 8–12 L/min), and random stagnation times that went up to 14 days. The water was also non-chlorinated, but originating mostly from untreated groundwater (95%) with a minor addition of pre-treated spring water (5%, UV disinfection, slow sand filtration) (Supplementary Table S1B).

### Sample Handling and Processing

**FIGURE 1:**
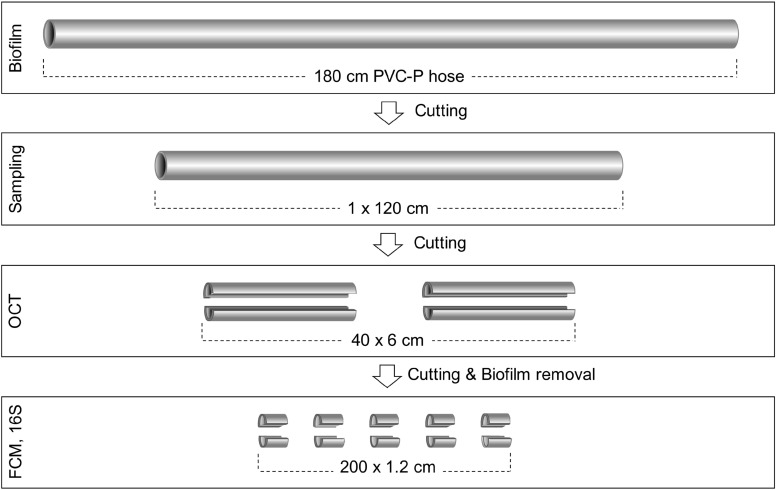
Experimental setup and sample processing. The control hose was horizontally aligned and flushed twice daily. The real hose hung vertically with a bend at the lower end and was used regularly (uncontrolled). The sampling strategy was identical for both hoses. A 120 cm section of each hose was extracted, then cut in 6 cm sections, horizontally bisected and imaged with optical coherence tomography for biofilm thickness. The 6 cm pieces were then cut into 1.2 cm sections followed by biofilm removal, which was then analyzed with flow cytometry for total cell counts and 16S rRNA amplicon sequencing for community analysis.

Both hoses were processed, sampled, and analyzed in the same way (Figure 1). For the control hose, 120 cm from the middle part were sampled for biofilm characterization, while for the real hose, 20 cm from the beginning of the hose (i.e., from the water inlet onward) were removed and the following 120 cm piece was sampled. The collected 120 cm pieces were each separately dissected into 20 × 6 cm pieces, which were then bisected into top and bottom sections. Optical coherence tomography (OCT) was used for imaging and quantifying biofilm structure and thickness (see section “Biofilm Analysis With Optical Coherence Tomography”). Following this, each piece was cut into 5 × 1.2 cm sections and biofilms were removed by brushing each of them separately with an electric toothbrush (Oral-B^®^, Advanced Power) into a total volume of 10 mL of 0.2 μm filtered bottled water (Evian, France). For this, each section was covered with 5 mL of filtered water in a petri dish and brushed for approximately 45 s, depending on the stickiness of the biofilm matrix. The remaining 5 mL were used to remove residuals of biofilm from the toothbrush head and from the surface of the petri dish (approximately 20 s brushing) and transferred to the sample tube. The 10 mL biofilm suspension was then needle sonicated to disrupt cell clusters (Sonopuls HD 2200, Bandelin Sonorex, Rangendingen, Germany). The needle was submerged to the upper third of the sample volume and sonication occurred for 30 s, with 5 × 10% pulses, and 40% power. The biofilm suspensions were measured with flow cytometry (FCM) to quantify total cell concentrations (TCC; see section “Flow Cytometry for Determining Total Bacterial Cell Concentrations”). Finally, biofilm suspensions were filtered for DNA analysis (see section “Community Analysis by 16S rRNA Gene Sequencing”). For all sampling steps, pieces were randomized to minimize the impact of processing errors.

For data analysis, the terminology “cm-sections” refers to the 1.2 cm sections, representing a total of 200 cm-sections per experimental hose. Furthermore, for bacterial cell concentrations and the analysis of sequencing data, units were converted to values per cm^2^ to make results more comparable within this study and to other studies.

### Biofilm Analysis With Optical Coherence Tomography

For characterizing the structure, biofilms were imaged using a Spectral Domain OCT Imaging System (930 nm, OCT System Ganymede, Thorlabs GmbH, Dachau, Germany), with an axial detection limit of 4.4 μm. The 6 cm pieces were horizontally aligned and covered with a thin layer of 0.2 μm filtered water for optimal imaging. Along the length of each piece, images of 2 mm (length) × 1 mm (height) were captured, which equals 30 images per piece or 1’200 per hose, respectively. The Advanced Positioning Technology (Thorlabs’ APT^TM^) Software was used to move the pieces in distinct steps of 2 mm without disrupting the alignment. Biofilm thickness was then determined using an analysis software in MATLAB^®^ (Version R2016b) which has previously been reported by Derlon and colleagues (Derlon et al., 2012). First, img files were translated into. tif images. Second, the interface between hose surface and biofilm was detected (gray-scale gradient analysis). In case of inaccurate detection of the interface, a black line was drawn manually using ImageJ (Version *1.50i*). Finally, these interfaces were used to create binary images, which were used for further image analysis. Potential problems that arose during image processing where solved as follows: (1) Detached biofilm structures floating around were creating artificially high values for biofilm thickness. For correction, these parts were masqued manually with black boxes (ImageJ). (2) If no clear detectable line was indicating the biofilm-water interface, it could result in wrong values for minimal biofilm thickness. For this, white lines were drawn manually indicating the biofilms surface (ImageJ). For better comparison between the different quantitative measurements, average thickness values were used for combined 1.2 cm sections (equals 6 images per section).

### Flow Cytometry for Determining Total Bacterial Cell Concentrations

Total bacterial cell concentrations (TCC) were quantified for each 1.2 cm section by FCM. Sample preparation, measurements, as well as data analysis were performed as described elsewhere (Prest et al., 2013). First, biofilm suspensions were diluted 1:10 (control hose) or 1:100 (real hose) respectively, with 0.2 μm filtered bottled water. Second, samples were stained with 10 μL/mL SYBR^®^ Green I (SG, Invitrogen AG, Basel, Switzerland; 100× diluted in Tris buffer, pH 8) to detect TCC. Finally, samples were incubated at 37°C for 10 min and then measured using a BD Accuri C6^®^ flow cytometer (BD, Belgium), with an instrumental threshold set at 800 (FL1-H) and a volume of 50 μL measured at a high flow velocity of 66 μL/min. For analysis, one gate was applied for all samples.

### Community Analysis by 16S rRNA Gene Sequencing

Prior to DNA extraction, biofilm suspensions were concentrated on 0.22 μm polycarbonate Nucleopore^®^ membrane filters (Ø 47 mm, Whatman, Kent, United Kingdom), using sterile bottletop filter units attached to a vacuum pump (vacuubrand 2c, Wertheim, Germany). DNA filters were immediately frozen in liquid nitrogen and stored at −20°C until DNA extraction.

#### DNA Extraction

DNA extraction was performed according to the protocol of the DNeasy PowerWater^®^ Kit (Qiagen, Hilden, Germany). Extracts were stored at −20°C until 16S rRNA gene amplification for sequencing.

#### 16S rRNA Gene Amplification and MiSeq Sequencing

For 16S rRNA gene sequencing, the V3-V5 region of the gene was amplified by polymerase chain reaction (PCR) using the primers Bakt_341F and Bakt_805R (Klindworth et al., 2013). First, DNA was quantified with the Qubit^TM^ DNA Broad Range Assay in duplicates, using the Spark^®^ 10M Multimode Microplate Reader (Tecan, Switzerland). The amount of DNA was normalized between all samples (1 ng) and primers were added in a final concentration of 0.3 μM (Supplementary Table S2A). After amplification, samples were purified with the Agencort AMPure XO System (Beckman Coulter, Inc., Bera, CA, United States), followed by the annealing of specific sequencing Nextera XT v2 Index Kit adapters (Illumina) to the generated amplicons via Index PCR (Supplementary Table S2B). Purified products were again quantified and the base pair (bp) length was verified with the High Sensitivity D1000 ScreenTape system (Agilent 2200 TapeStation), identifying an average library size of 569 bp. Each sample was normalized to 2 nM (10 mM Tris, pH 8.0), followed by pooling 10 μL of each, and a last quantification to ensure the final concentration. The Illumina MiSeq platform was used for paired-end 600 cycle sequencing with 10% PhiX serving as a control in the sequencing run (Illumina: Technical Note on PhiX Control). For amplification and sequencing, a distinct number of samples was processed in duplicates to verify the reliability and reproducibility of sequencing data. Also, a negative control (amplification of PCR grade water) as well as a positive control (Self-made MOCK community: *Burkholderia xenovorans*, *Bacillus subtilus*, *Escherichia coli*, *Micrococcus luteus*, *Pseudomonas protegens*, *Paenibacillus sabinae*, *and Streptomyces violaceoruber*) were incorporated. In the course of sample processing, some biofilm sections needed to be excluded due to low quantities of extracted DNA, poor amplification, or poor number of reads after sequencing. Data on community composition was generated in collaboration with the Genetic Diversity Centre (GDC), ETH Zurich.

### Sequencing Data Processing and Analysis

16S rRNA amplicon sequence data were processed following a distinct pipeline. First, data quality was evaluated (Supplementary Table S3, step A). Second, read ends were trimmed and merged (Supplementary Table S3, step B). Third, *in silico* PCR was performed and primer sites trimmed (Supplementary Table S3, step C). Then, sequences were filtered based on their quality and size range (Supplementary Table S3, step D). Finally, amplicon sequence variants (ASV) were established and taxonomically assigned. In contrast to the classic 97% identity clustering method (Schloss and Westcott, 2011), sequences were clustered by an ASV approach using UNOISE3 (Edgar, 2017). Unoise3 includes a sequencing error correction and chimaeral removal. The predicted biological sequences (i.e., ASV) are called zero-radius operational taxonomic units (ZOTUs). Although ZOTUs are valid operational taxonomic units (OTUs) the number is usually inflated. The reason might be the fact that early PCR errors cannot be detected and are therefore leading to very similar amplicons. For this reason, we additionally clustered the ZOTUs at different identity levels (99, 98, and 97%). For the taxonomic assignment predictions, the Silva 16S database (v128) in combination with the SINTAX classifier was used with a cut-off of 0.9. Attributed classifications for DNA sequences were ultimately verified using the NCBI platform. Data analysis was performed using R (Version 3.3.0) and RStudio (Version 1.1.477) with the R package ggplot2 (Version 2.2.1), vegan (version 2.4.5) and the Bioconductor “phyloseq” (Version 1.16.2). See information on the R-code in Supplementary Information.

#### Data Availability

DNA sequencing data is available via the Sequence Read Archive (SRA) of the National Center for Biotechnology Information (NCBI): Accession number PRJNA554997.

### Scanning Electron Microscopy

Ten centimeters from the beginning and end of each hose were immediately prepared for scanning electron microscopy. For this, biofilms were fixed with 2.5% Glutaraldehyde in Cacodylate buffer (0.1 M, pH 7.2) at room temperature for 60 min and stored in Cacodylate buffer at 4°C afterwards. Final preparation and imaging was done by the Center for Microscopy and Image Analysis (University of Zurich).

## Results

We analyzed in detail biofilms that formed inside two identical shower hoses under controlled use and real use conditions, with both exposed to non-chlorinated warm water during approximately 12 months. The purpose of this study was to assess the degree of spatial heterogeneity within each individual biofilm by high-resolution sampling, with the communities developing under supposedly uniform (control hose) or more variable (real hose) environmental conditions. In Figure 1, the two longitudinal halves of each hose are categorized as *top* and *bottom*, reflecting the actual spatial orientation of the control hose in the laboratory setup. The real hose was used vertically in a shower, hence the longitudinal *top* and *bottom* do not represent any specific orientation. Data from 200 biofilm sections was analyzed on various scales (from μm–m) for each individual hose. Here, *large-scale* refers to the complete hose (i.e., the 120 cm piece of hose). *Small-scale* refers to the differences between adjacent 1.2 cm-sections.

### Biofilm Development Under Controlled Conditions

**FIGURE 2:**
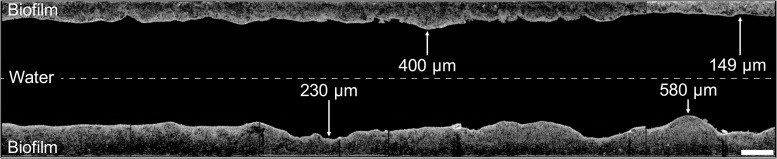
Visualization of the control hose biofilm imaged with optical coherence tomography. Images (2 mm length) were combined to illustrate the biofilm structure and thickness of 1.2 cm-sections, showing a representative example of the shower hose biofilm. The hose was static and horizontally aligned, thus top and bottom in this image represent the actual orientation of the biofilm. The space between the top and bottom sections is not to scale. Scale bar: 500 μm.

**FIGURE 3:**
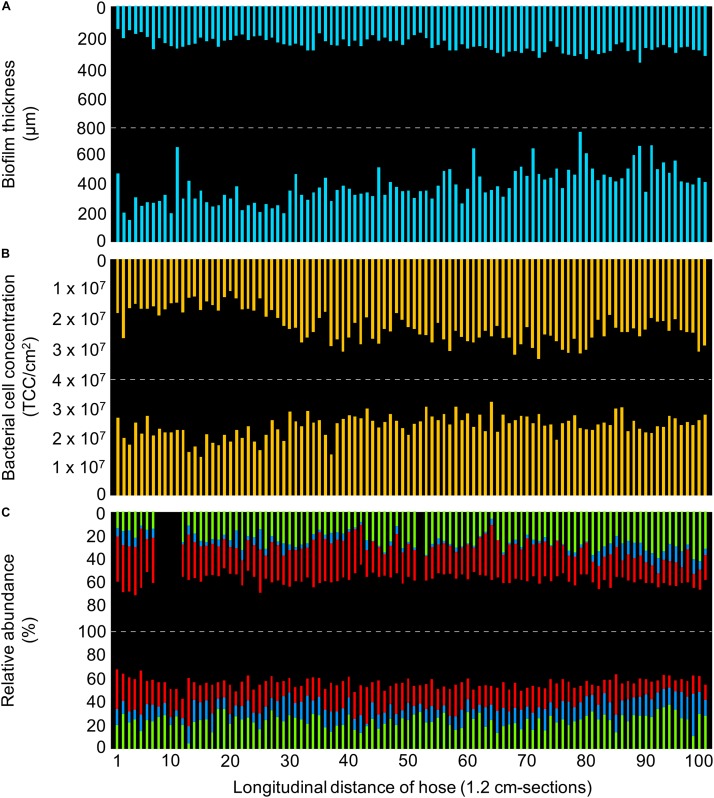
Detailed characterization of the control hose biofilm, with bars representing individual sections of 1.2 cm. **(A)** Biofilm thickness measured with optical coherence tomography. **(B)** Bacterial cell concentrations measured with flow cytometry. **(C)** Community composition measured with 16S rRNA gene sequencing, showing the relative abundance of the three most abundant taxa (green: Cytophagaceae; blue: TM6_[Dependentiae]; red: *Bradyrhizobium* spp.). Data gaps resulted from insufficient DNA amplification.

A visibly thick biofilm established on the inner surface of the control hose during 12 months of twice-daily warm water flushing (Figures 2, 3A). The entire biofilm of the 120 cm piece of the hose contained a total of 4.7 × 10^9^ bacteria, at an average distribution of 2.4 ± 0.5 × 10^7^ cells/cm^2^ (*n* = 200) (Figure 3B), with the microbial community being dominated by only few taxa (Figure 3C).

#### Structure: Thickness Varied on μm-Scale

The biofilm topography was sinuous, with uneven protrusions and depressions resembling hills/dunes (Figure 2). The averaged thickness of 1.2 cm-sections ranged between 150–750 μm with an overall average of 319 ± 111 μm (*n* = 200) (Figure 3A and Supplementary Figure S2). On large-scale, the biofilm was significantly thicker at the bottom (386 ± 117 μm, *n* = 100) compared to the top (252 ± 44 μm, *n* = 100) (*t*-test, *p* < 0.05). Moreover, biofilm thickness increased notably over the length of the 120 cm piece following the flow direction; approximately 100% in the top (linear regression with R^2^ = 0.43) and 255% in the bottom (linear regression with R^2^ = 0.40). This amounts to an average increase of 0.83 μm/cm (top) and 2.13 μm/cm (bottom). In addition to the spatial trend, variability in biofilm thickness was already evident on small-scale. Adjacent cm-sections of the top varied 11.7 ± 8.9% (*n* = 99), ones in the bottom varied even more with 23.9 ± 28.5% (*n* = 99), with the standard deviations suggesting higher variation/heterogeneity throughout the bottom part of the hose. On an even smaller scale (i.e., μm-scale), variations of up to 50% could be observed (Figure 2). In addition to the assessment of structural heterogeneity, two-dimensional thickness data could be used to roughly reconstruct three-dimensional characteristics. Here, the average biofilm volume, calculated from the average thickness data, was 2.5 ± 0.4 × 10^10^ μm^3^/cm^2^ (*n* = 100) in the top and 3.9 ± 1.2 × 10^10^ μm^3^/cm^2^ (*n* = 100) in the bottom part of the hose.

### Numbers: Bacteria Account for Only a Small Fraction of the Biofilm Volume

Total cell concentrations (TCC) of 1.2 cm-sections ranged between 1.1–3.4 × 10^7^ cells/cm^2^. Interestingly, average TCC values were the same at the top (2.3 ± 0.5 × 10^7^ cells/cm^2^, *n* = 100) and at the bottom (2.4 ± 0.4 × 10^7^ cells/cm^2^, *n* = 100), in stark contrast to the thickness data presented above. Correlations between TCC and biofilm thickness were weak, but higher for the top (R^2^ = 0.27; Pearson correlation *r* = 0.5) compared to the bottom biofilm (R^2^ = 0.07; *r* = 0.3). On large-scale, linear regressions suggest an increasing trend in TCC along the length of the hose for both top (R^2^ = 0.36) and bottom (R^2^ = 0.17). However, this trend is mainly driven by lower concentrations in the first 30 cm-sections of the control hose, with on average 34% lower concentrations in the top and 17% in the bottom part compared to the rest of the hose (Figure 3B). Fluctuations on small-scale, i.e., between adjacent cm-sections, were similar in top (14.9 ± 11.6%, *n* = 99) and bottom (14.7 ± 13.0%, *n* = 99). The combination of the TCC data and an estimated average cell volume of 0.3 μm^3^ [calculation based on average cell size from SEM imaging, Supplementary Figure S3A; comparable to Heldal et al. (1994)] allows the calculation of total bacterial cell volume in the biofilm, which was on average 6.8 ± 1.6 × 10^6^ μm^3^/cm^2^ (*n* = 100) in the top and 7.0 ± 1.1 × 10^6^ μm^3^/cm^2^ (*n* = 100) in the bottom. This, in turn, allows the calculation of the relative contribution of bacterial cell volume to the overall biofilm volume (V_cells_:V_biofilm_), which was notably small with approximately 0.03 ± 0.01% (*n* = 100) for the top and 0.02 ± 0.01% (*n* = 100) for the bottom biofilm.

#### Microbiome: Biofilm Community Dominated by Only Few Taxa

The overall biofilm community comprised 384 ZOTUs (henceforth referred to as taxa). On large-scale, ordination by non-metric multidimensional scaling, based on the Bray-Curtis dissimilarity, showed a clear trend in sample clustering in the control hose (Supplementary Figure S4A). Here, orientation (i.e., top vs. bottom) accounted for 22% of community variations (adonis, *p* < 0.001). Following this, taxa richness (S) was higher in the top (S = 335) compared to the bottom (S = 288), both with an Evenness index (J′) of 0.4. On small-scale, richness ranged from 55 to 92 taxa/cm-section (J′ = 0.5–0.6), with on average 72 ± 6 taxa/cm-section (*n* = 95) in the top and 67 ± 6 taxa/cm-section (*n* = 100) in the bottom. In addition, richness showed variations between adjacent cm-sections of 9 ± 7% (*n* = 92) in the top and 7 ± 6% (*n* = 99) in the bottom. Regarding beta-diversity, Bray-Curtis revealed compositional dissimilarities in the communities of adjacent cm-sections between 0.05–0.38 (average 0.15 ± 0.06, *n* = 191), arguing in favor of a rather similar community composition along the length of the biofilm on small-scale. Interestingly, only few dominant taxa (i.e., taxa with at least 1% of the total number of reads) made up the majority of the community composition. In fact, the 10 most dominant taxa accounted for 89.3% of the total biofilm community (Supplementary Table S4), covering 90.0% in the top and 89.6% in the bottom community composition of the hose. Moreover, the three most dominant taxa even made up 56.7% of the community and were identified as (1) an uncultured genus of the family Cytophagaceae (24.7%, Figure 3C, green), (2) *Bradyrhizobium* spp. (23.4%, Figure 3C, red), and (3) an uncultured representative of the phylum TM6_[Dependentiae] (9.6%, Figure 3C, blue). The remaining seven dominant taxa were identified as *Dechloromonas* spp., *Denitratisoma* spp., *Sediminibacterium* spp., *Brevifollis* spp., *Ohtaekwangia* spp., and *Rhodobacter* spp., as well as another member of the family Rhodobacteraceae which could not be identified further (Supplementary Table S4). Due to the dominance of similar if not the same taxa in top and bottom, a comprehensive analysis of potential spatial variations over the length of the hose for shared taxa was possible. On large-scale, Cytophagaceae and *Bradyrhizobium* spp. had a negative correlation in both top (R^2^ = 0.67) and bottom (R^2^ = 0.45) (Supplementary Figure S5). Also, repetitive fluctuations along the length of the hose were identified. For example, the detection of Cytophagaceae showed an increase in its relative abundance from 19.9 ± 3.4% (*n* = 11) to 25.8 ± 3.1% (*n* = 11) following sections 63–84 in the bottom (Figure 3C, green). On small-scale, sections of localized heterogeneity were detected. For example, TM6_[Dependentiae] showed a clear difference in its abundance between sections 70–80 and 81–91 in the top of the hose; with an increase in relative abundance from 2.5 ± 1.3% (*n* = 11) to 10.6 ± 2.2% (*n* = 11) (Figure 3C, blue). Overall, correlations between the relative abundance of specific taxa and (1) thickness (R^2^ < 0.14), (2) TCC (R^2^ < 0.1), or (3) richness (R^2^ < 0.2) were weak.

### Biofilm Development Under Real Conditions

**FIGURE 4:**
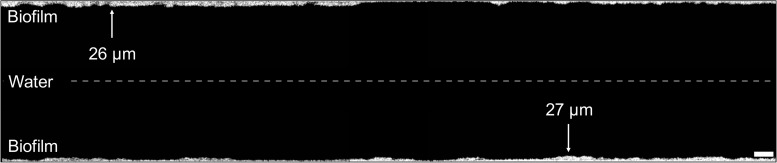
Visualization of the real hose biofilm imaged with optical coherence tomography. Images (2 mm length) were combined to illustrate the biofilm structure and thickness of a mm section, showing a representative example of the shower hose biofilm. Distance between top and bottom sections is not to scale. Scale bar: 200 μm.

**FIGURE 5:**
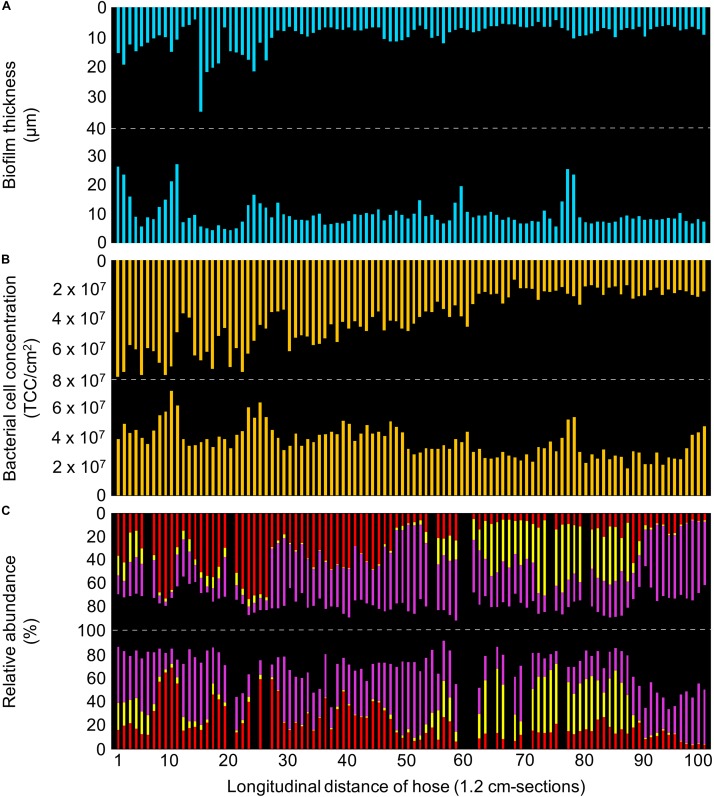
Detailed characterization of the real hose biofilm, with bars representing individual sections of 1.2 cm. **(A)** Biofilm thickness measured with optical coherence tomography. **(B)** Bacterial cell concentrations measured with flow cytometry. **(C)** Community composition measured with 16S rRNA gene sequencing, showing the relative abundance of the three most abundant taxa (red: *Bradyrhizobium* spp.; yellow: *Altererythrobacter* spp.; purple: *Caulobacter* spp.). Data gaps resulted from insufficient DNA amplification.

A comparatively thin biofilm established on the inner surface of the real hose during 12 months of random usage and handling (Figures 4, 5A). The 120 cm piece of hose contained a combined total of 7.6 × 10^9^ bacteria at an average distribution of 3.8 ± 1.4 × 10^7^ cells/cm^2^ (*n* = 200) (Figure 5B). The bacterial community composition was also dominated by only few taxa (Figure 5C), comparable to the control hose. While the data is visualized as longitudinal top and bottom (Figure 5) this does not represent the actual orientation of use, but rather two opposite sides of the hose. Therefore, samples of different orientation (i.e., *top* vs. *bottom*) were not analyzed separately.

#### Structure: A Comparatively Thin Biofilm Developed Inside the Real Hose

The real hose biofilm was considerably thinner than the control hose biofilm, often below the OCT detection limit (∼4 μm), but also showing uneven protrusions and depressions throughout (Figure 4 and Supplementary Figure S3B). The average thickness per 1.2 cm-section ranged from 4.3 μm up to 35.9 μm with an overall average of 9.8 ± 4.6 μm (*n* = 200) (Figure 5A and Supplementary Figure S6). On large-scale, the biofilm was notably thicker in the first ∼30 cm-sections (26.0 ± 8.9 μm, *n* = 27) (i.e., lower end of the vertically hanging hose) compared to the rest of the hose (17.3 ± 3.8 μm, *n* = 73). On small-scale, we observed considerable heterogeneity between adjacent cm-sections (average 23.4 ± 54%, *n* = 198; Figure 5A), with the average being comparable to those of the control hose biofilm. This structural heterogeneity is evident on even smaller, μm-scale, where variations of up to 74% in biofilm thickness could be identified (Figure 4). Consistent to the control hose, thickness data was used to calculate the approximate average biofilm volume, which was 9.8 ± 4.6 × 10^8^ μm^3^/cm^2^ (*n* = 200).

#### Numbers: Bacterial Cell Concentrations Are in the Same Magnitude as in the Control Biofilm

Total cell concentrations of 1.2 cm-sections ranged between 1.5–8.1 × 10^7^ cells/cm^2^ (Figure 5B), thus being in the same order of magnitude as the control hose biofilm while overall covering a broader range. Interestingly, correlations between TCC and biofilm thickness were higher in the real hose (R^2^ = 0.37; *r* = 0.6) compared to the control hose biofilm (above). On large-scale, linear regression showed an ongoing decreasing trend over the length of the entire hose (R^2^ = 0.73). Small-scale heterogeneity between adjacent cm-sections was on average 17.2 ± 15.2% (*n* = 198), thus comparable to results from the control hose biofilm. The combination of TCC and an average cell volume (0.3 μm^3^) accounted in this hose biofilm for an average bacterial cell volume of 1.1 ± 0.4 × 10^7^ μm^3^/cm^2^ (n = 200). This, in turn, allows the calculation of the relative contribution of bacterial cell volume to the overall biofilm volume (V_cells_:V_biofilm_) which was about 1.2 ± 0.5% (*n* = 200) and therefore considerably higher than in the control hose.

#### Microbiome: Community Dominated by Different Taxa Than the Control Hose Biofilm

On large-scale, no significant heterogeneity in the community composition was caused by the orientation of the hose (Supplementary Figure S4B), as was expected due to regular movements and re-orientation of the hose during usage. Interestingly, the community compositions of the control and the real hose biofilms showed clear differences when illustrating Bray-Curtis dissimilarities (Supplementary Figure S4C). Here, the two different experiments (biofilm growth under laboratory conditions vs. under realistic conditions) accounted for 65% of community variation (adonis, *p* < 0.001). It should be noted that input water varied between these two locations, in addition to the differences in operation (Supplementary Table S1). Regarding alpha-diversity, however, taxa richness was comparable to the control hose biofilm, with 341 taxa and an Evenness index of 0.4. On small-scale, richness ranged from 37 to 119 taxa/cm-section (J′ = 0.3–0.6), with an average of 64 ± 14 taxa/cm-section (*n* = 183). Also, random fluctuations between adjacent cm-sections showed variations in richness, with 15 ± 14% (*n* = 165). These were less pronounced compared to ones in the control hose. Bray-Curtis dissimilarity showed variations in beta-diversity of adjacent sections, ranging from 0.04 to 0.55 (average: 0.18 ± 0.1, *n* = 169), and again highlighting a similar pattern in community composition heterogeneity as the control hose biofilm. Moreover, only few taxa dominated the community composition (i.e., covering at least of 1% of the total number of reads), which was consistent with the control hose biofilm. Here, the 10 most dominant taxa accounted for 90.4% of the entire community composition (relative abundance; Supplementary Table S5). Comparable to the control hose biofilm, a comprehensive analysis of potential spatial variations over the length of the hose was conducted for dominant shared taxa. The three most abundant taxa made up for 73.2% of the community and were identified as (1) *Caulobacter* spp. (34.7%, Figure 5C, purple), (2) *Bradyrhizobium* spp. (24.2%, Figure 5C, red), and (3) *Altererythrobacter* spp. (14.2%, Figure 5C, yellow). The remaining seven dominant taxa were identified as *Brevibacterium* spp., *Bosea* spp., *Bdellovibrio* spp., *Sphingomonas* spp., *Rhodobacter* spp., as well as two members of the family Chitinophagaceae and one representative of the phylum Cyanobacteria (Supplementary Table S5). Consistent with the analysis of the control hose biofilm data, spatial variations for the three most dominant taxa were analyzed. On large-scale, a negative correlation between *Caulobacter* spp. and *Bradyrhizobium* spp. was identified (R^2^ = 0.34; Supplementary Figure S7). Also, repetitive fluctuations in relative abundances were observed. For example, *Caulobacter* spp. increased in its abundance from sections 77 to 88 (27.2 ± 5.9%, *n* = 11) to the following sections 89–99 (54.4 ± 13.0%, *n* = 11), corresponding to an increase of 27% (Figure 5C, purple). On small-scale, obvious localized heterogeneity of *Altererythrobacter* spp. was detectable, which in fact was more pronounced than in the control hose biofilm. Here, the relative abundance of 18.3 ± 4.5% (*n* = 11) decreased to an average of 8.3 ± 4.2% (*n* = 11) within the range of sections 79–100 (Figure 5C, yellow). Overall, correlations between taxa relative abundance and (1) thickness, (2) TCC and/or (3) richness were mostly poor (R^2^ < 0.2), with an exception in the relative abundance of *Bradyrhizobium* spp. which positively correlated with TCC (R^2^ = 0.37).

## Discussion

Microbial heterogeneity within large, but connected, ecosystems was previously characterized in both natural (Langenheder et al., 2017; Liu et al., 2018) and engineered (Liu et al., 2013; Roeselers et al., 2015) ecosystems. The purpose of this study was to assess spatial heterogeneity within a confined engineered ecosystem (120 cm of flexible shower hose) in detail by characterizing biofilm structure, cell numbers, and microbial community composition on various scales (from μm to m) with high-resolution sampling. Ultimately, this can allow for a better understanding of the driving forces of biofilm formation and localized biofilm heterogeneity in building plumbing systems and the broader implications of such heterogeneity on biofilm sampling and analysis strategies.

### Dispersal and Selection Drive Homogenous Biofilm Assembly Under Otherwise Uniform Environmental Conditions

Microbial heterogeneity within drinking water pipes was previously ascribed to variations in material properties, e.g., surface structure and adhesion characteristics (Pasmore et al., 2002), as well as chemical and physical characteristics of the water (e.g., nutrients, pH) (Lehtola et al., 2004), flow velocity, and shear stress (Lehtola et al., 2006; Moreira et al., 2013). In our study, we presumed uniformity in all these environmental variables along the length of the control hose, and we hypothesized that a biofilm, formed under such spatially uniform environmental conditions, would be homogeneous in terms of structure, cell numbers, and community composition.

Biofilm development was mainly driven by two ecological processes, namely *dispersal* of cells from the source water and *selection* based on growth (Hubbel, 2006; Kinnunen et al., 2016). The repetitive introduction of the same microbial community along the length of the hose through twice-daily flushing events allowed for a homogeneous dispersal of bacteria from the water to the biofilm, and thus an initially uniform bacterial distribution (numbers and community composition) throughout the hose. However, we believe that initial dispersal-driven assembly was less important for the final biofilm composition than niche assembly (i.e., selective growth). In fact, dispersal assembly alone did not nearly account for the biofilm TCC measured after one year (on average 2.4 × 10^7^ cells/cm^2^; Figure 3B), based on the water phase TCC (∼10^5^ cells/mL^26^). As Swiss tap water is usually carbon limited (Lautenschlager et al., 2010), biofilm growth on synthetic polymeric pipe surfaces is primarily driven by the organic carbon migrating from the material (Bucheli-Witschel et al., 2012; Connell et al., 2016). Biodegradable carbon compounds that migrate from flexible plastic materials into the (drinking) water phase (e.g., flexibilizers, plasticizers) were shown to increase microbial growth rates and yields (Zhang and Liu, 2014; Wen et al., 2015). Several previous studies quantified migrating organic carbon as the main carbon source for microbial growth (e.g., Bucheli-Witschel et al., 2012; Proctor et al., 2016). Here we assumed, but did not specifically quantify, that the migration of biodegradable carbon compounds is homogenous along the length of a 120 cm shower hose. An assumed uniform migration of these biodegradable carbon compounds should impact biofilm development equally throughout the length of the hose, thus allowing for a homogeneous distribution in cell concentrations but also community compositions (Figures 3B,C). In addition, since these migrating compounds are the predominant carbon sources in this environment, a specific niche is created that results in a selective pressure within the developing microbial community (Vandermeer, 1972). Several studies showed that growth on specific substrates results in the selection of specific taxa even when starting with complex starting communities (Proctor et al., 2016; Goldford et al., 2018; Rivett and Bell, 2018), and also indicated lower richness in biofilms compared to planktonic communities (Henne et al., 2012; Liu R. et al., 2014). This selective effect was clearly detectable in our study by a considerable decrease in diversity in the biofilm communities on large-scale. While the initial tap water microbiome was highly diverse with approximately 5’000 different taxa (data not shown), individual biofilms showed a lower total diversity with <400 taxa. In fact, the three most abundant taxa accounted for the majority of the biofilm communities (Figures 3C, 5C).

### Different Variability in Environmental Conditions Between Similar but Disconnected Ecosystems Result in Microbial Heterogeneity

Biogeographical heterogeneity is commonly observed in seemingly similar environments that are not physically connected. For example, differences in microbial communities were observed when comparing different drinking water treatment plants, individual water meters (Roeselers et al., 2015), or shower hoses (Proctor et al., 2016). Also, on a laboratory scale, biofilms that developed from an identical starting community were dominated by different taxa, which was attributed to the availability of different carbon sources with otherwise identical environmental conditions (Haagensen et al., 2015; You et al., 2015; Goldford et al., 2018). These examples emphasize that even though environmental conditions are assumed to be similar between two (disconnected) systems (e.g., treatment plants, water meters; Roeselers et al., 2015), already relatively small differences can result in microbial variations, i.e., heterogeneity.

Our study focused on heterogeneity at high spatial resolution within an individual biofilm formed on a single hose (i.e., single environment). The inclusion of a second hose biofilm from an environment with arguably more variability in environmental conditions expanded the broader applicability of the findings to other systems. Both setups comprised identical material but showed differences in usage and incoming water compositions. As a result, the extent of the individual small-scale heterogeneity was different between the two biofilms, but also considerable differences between the two similar but disconnected (i.e., individual) ecosystems were detected. Firstly, the biofilm of the real hose was ten-fold thinner than the one of the control hose (Figures 2, 4). It was shown before that higher flow rates result in thinner biofilm structures compared to slow flow conditions (van Loosdrecht et al., 1995). As this was the case for the control (0.3 L/min) and the real (10–12 L/min) hose setups, it poses one plausible explanation for the observed difference in biofilm thickness. Despite these differences in thickness, TCC were comparable between the two biofilms, interestingly suggesting a similar growth potential and/or total carrying capacity. Secondly, the overall biofilm communities of both biofilms (control and real hose) were dissimilar (Supplementary Figure S4C). One reason for these inter-hose variations is the different source waters. With the installations being located in two cities, water sources, treatment, and distribution were different and therefore resulted in different bacterial community compositions (Supplementary Tables S4, S5; Pinto et al., 2012; Liu et al., 2013; Ma et al., 2017). Also, in the real hose setup, a mixture of hot and cold tap water was used while the control hose was only flushed with water from the hot water line, again providing different community compositions within the waters (Henne et al., 2013). Consequently, dispersal-driven assembly was different between the two hoses and allowed for different organisms to settle, attach, and grow.

Comparing the dominant taxa between control and real hose revealed only little consensus between the biofilms. For example, only one out of ten taxa were identical on genus level (*Bradyrhizobium* spp.) and only two were similar on family level (Bradyrhizobium, Chitinophagaceae) (Supplementary Tables S4, S5 ). It was previously shown that the availability of different nutrients enables distinct phylogenetic families to outgrow others in a given ecosystem (You et al., 2015; Goldford et al., 2018), based on the ability and efficiency of metabolizing these. In both the control and the real hose setup, migration from the flexible plastic material provided the major carbon source, allowing bacteria that are capable of metabolizing these compounds to outcompete others (niche assembly, Hubbel, 2006). The comparison of these two similar but disconnected ecosystems illustrates (1) how environmental conditions shape heterogeneity (e.g., impact of flow rate and dispersal), but also (2) how a dominant carbon source (e.g., migrated from flexible PVC-P) results in comparably low diversity in two otherwise distinct biofilm communities (Figures 3C, 5C). While these differences were obvious on a taxonomic level, no metabolic analyses were performed (e.g., enzyme expressions). In fact, despite a distinct taxonomic assignment, taxa might still perform similar metabolic actions (Ji et al., 2017).

The differences between the control and the real hose were interesting. However, these hoses represent single examples from each environment (laboratory and real-use conditions) and thus provide insufficient replication for (1) representing biofilms of these environments in general and (2) for drawing definitive conclusions on the role of the environment on biofilm formation. Rather, the focus of this study was on the small-scale variations within the biofilms of each hose.

### Small-Scale Differences in Environmental Variables Drive Heterogeneity Within a Connected Ecosystem

Heterogeneity in microbial assemblages of connected ecosystems has been widely attributed to localized variations in environmental conditions (Hou et al., 2017; Langenheder et al., 2017). Patchiness (i.e., heterogeneity) has even been described within individual biofilms on small-scale, i.e., in systems with apparent uniform conditions (van Loosdrecht et al., 1995; Lowery et al., 2017). Overall, conditions in the control hose setup were kept as uniform as possible. However, the horizontal alignment introduced a distinct difference between the bottom and the top part as a result of gravity. Gravity was previously identified as a driver for heterogeneity along a radial-spatial orientation due to particle deposition (Liu et al., 2017) and the rising of air bubbles (Jang et al., 2017). It is probable that the deposition of inorganic particles, which occurred especially during stagnation, over the course of one year of operation contributed to the thicker biofilm in the bottom part of the control hose without significantly affecting the cell concentration (Figures 3A,B). In addition, biofilm sloughing by air bubbles during flow, potentially contributed to a thinner and more variable biofilm structure in the top biofilm compared to the bottom (Figure 3A; Jang et al., 2017).

In the real hose, which was installed vertically, gravity obviously impacted biofilm thickness differently, with particles likely accumulating in the lower bend. Here, we observed clear heterogeneity with thicker patches of biofilm in the lower section and a continuously decreasing gradient in TCC along the length of the hose (Figures 5A,B). In addition, the orientation of the real hose also probably impacted flow dynamics (i.e., with a lower bend). Changes in flow velocity (Douterelo et al., 2013) and turbulence (Tsagkari and Sloan, 2018) were previously shown to impact community composition and biofilm thickness.

In both the control and the real hose biofilm, community composition showed heterogeneity on both large- and small-scale. For example, the relative abundance of some of the most dominant taxa changed on large-scale along the length of the hose, gradually as well as fluctuating. On small-scale, localized heterogeneity was observed for dominant taxa of both control and real hose biofilms (Figures 3C, 5C), being more pronounced in the latter. Previous research showed patchiness (i.e., small-scale heterogeneity) in biofilms due to factors like predation and grazing (Huws et al., 2005; Derlon et al., 2012), successive growth, e.g., based on by-products (Elias and Banin, 2012), oxygen availability and mass transport (de Beer et al., 1994), variable strategies for colony expansion (Goldschmidt et al., 2017), competition and cooperation (Nadell et al., 2016; Rendueles and Velicer, 2016) and heterogeneity in nutrient gradients and growth dynamics (Sternberg et al., 1999; Kreft and Wimpenny, 2001). While any of these could be relevant, our analyses were not designed to untangle any one dominant factor.

### Practical Implications

The assessment/characterization of small-scale heterogeneity within individual biofilms allows us to draw several conclusions regarding sampling and analysis strategies on a broader scale. Sample size and the required number and spatial distribution of sampling points within a given system are some of the most critical issues when considering biofilm sampling strategies. Across disciplines, biofilm characterization is often limited by the accessibility of the relevant surface which necessarily results in diverse sampling approaches. Consequently, sample sizes in biofilm studies range from microscopic analysis on μm-scale (Batté et al., 2003; Mitri et al., 2015; Dal Co et al., 2019) to microbiome studies on single-digit cm-scale (Liu et al., 2012; Ma et al., 2018), up to specifically designed insertable coupons (e.g., 2.24 cm^2^; Deines et al., 2010; Fish et al., 2016; Douterelo et al., 2017) as well as whole pipe/hose sections of, e.g., up to 90 cm in length (Liu et al., 2017; Proctor et al., 2018). Our data highlights the importance of sample size and distribution, as any prevalent spatial heterogeneity influences the representativeness of a sample and therefore impacts conclusions that are drawn.

In the present study, the combination of individual results (i.e., 1.2 cm sections) allowed us to simulate larger sample sizes and to compare these results. For example, the average of ten adjacent samples provides the (theoretical) outcome of sampling the length of 12 cm as one single sample. It is obvious that a sampled biofilm area should be as large as possible to obtain a characterization as close as possible to the average of an entire system (Supplementary Figure S8). However, while sampling an entire biofilm may well be feasible for shower hoses (Proctor et al., 2016, 2018), this would not be realizable for large pipes or surfaces (Liu G. et al., 2014; Lührig et al., 2015). As soon as smaller area sizes are sampled, spatial heterogeneity [e.g., top/bottom caused by gravity (Liu et al., 2017; Figure 3A) or longitudinal (Potgieter et al., 2018; Figure 5B)] consequently requires multiple sampling points to capture the heterogeneity within one system, e.g., based on pipe orientation. The data shown in the present study encourages researchers to sample biofilms as representative as possible. Specifically, this means collecting biofilms either from large surface areas or from multiple, distributed small areas, to balance out small-scale heterogeneity. Moreover, we encourage biofilm researchers to both assess and illustrate the representativeness of their sample collection strategy when reporting.

While smaller sampling areas result in large deviations from the overall average (Supplementary Figure S8) and reduce the representativeness of one sample for an entire system, sampling on small-scale (μm-cm) is particularly valuable if the uniqueness of a system/biofilm is of interest. Our results showed that even if environmental conditions are assumingly uniform, heterogeneity can develop on small-scale in a biofilm. This emphasizes that a biofilm is very unlikely to be homogeneous and thus requires sampling at different locations. Biofilms and microbial communities have previously been compared to landscapes, i.e., environments consisting of spatial variations and showing complex ecological interactions (Turner, 2005; Battin et al., 2007). Here, variations in environmental conditions can, for example, be introduced by gradients on μm-scale (e.g., oxygen, pH, nutrients), which allow for the establishment of different micro-environments and ecological niches (de Beer et al., 1994; Schramm et al., 2000). With limitations in certain resources, bacteria need to adapt, cooperate, and/or compete, which ultimately results in selected bacterial clusters and a distinct spatial organization (Stewart and Franklin, 2008; Mitri et al., 2015; van Gestel et al., 2015; Schreiber et al., 2016). It is necessary to sample and analyze biofilms on very small scales to allow for the identification of this heterogeneity, and important as processes on such small scale ultimately shape large-scale pattern and effect ecosystem functioning (Guichard and Bourget, 1998; Singer et al., 2010).

## Conclusion

• High-resolution sampling of shower hose biofilms (200 samples/120 cm) in addition to detailed analysis on various scales (μm–m), enabled the assessment of small-scale spatial heterogeneity in biofilm structure, bacterial numbers, and community composition.
• A biofilm grown inside a flexible hose under controlled laboratory conditions, was likely uniformly exposed to processes such as dispersal, carbon migration, growth, and selection along its length. Accordingly, the respective biofilm was homogenous on large-scale, but showed notable localized heterogeneity on small-scale.
• A biofilm grown under real (i.e., uncontrolled) use conditions showed considerably more variations in all variables on both large- and small-scale, with particularly clear spatial fluctuations in the relative abundance of dominant taxa.
• The control hose biofilm was different to the real hose biofilm with respect to thickness and community composition, which was most probably influenced by different operational conditions and water sources. However, both hoses showed impressively low biofilm community diversity, which was attributed to the selective force of the migrating carbon from the flexible PVC-P hoses.
• In addition, our results show that the adequate biofilm sample size strongly depends on the research question: whether the small-scale uniqueness of an ecosystem is explored (μm- to cm-scale), or whether an average overview of an entire system is required (cm- to m-scale).

## Data Availability Statement

The datasets generated for this study can be found in the NCBI Sequence Read Archive (SRA) number PRJNA554997.

## Author Contributions

LN: experimental design, experimental work, data analysis, and manuscript writing. CP: experimental design, experimental work, and data analysis. J-CW: sequencing data processing and manuscript writing. FH: experimental design, experimental work, and manuscript writing.

## Conflict of Interest

The authors declare that the research was conducted in the absence of any commercial or financial relationships that could be construed as a potential conflict of interest.
