# Supplementary material for: Small-Scale Heterogeneity in Drinking Water Biofilms
Source: Front Microbiol. 2019 Oct 29;10:2446. doi: 10.3389/fmicb.2019.02446 (PMC6828615; doi:10.3389/fmicb.2019.02446)
Supplement: Supplementary file 1 [file Data_Sheet_1.PDF]

*Supplementary Material*

# Small-scale heterogeneity in drinking water biofilms

**Lisa Neu[1,2], Caitlin R. Proctor[1,3], Jean-Claude Walser[4], Frederik Hammes[1*]**

[1] Department of Environmental Microbiology, Eawag: Swiss Federal Institute of Aquatic Science and Technology, Dübendorf, Switzerland

[2] Department of Environmental Systems Science, Institute of Biogeochemistry and Pollutant Dynamics, ETH Zürich, Zürich, Switzerland

[3] Schools of Civil, Environmental and Ecological, Materials, and Biomedical Engineering, Purdue University, West Lafayette, Indiana, United States

[4] Genetic Diversity Centre (GDC), ETH Zürich, Zürich, Switzerland

**Table of contents**

| | |
|---|---|
| **Figure S1** | Temperature pattern in control hose during operation |
| **Figure S2** | Thickness values for control hose biofilm on 2mm-scale |
| **Figure S3** | Scanning electron microscopy images (control & real Hose) |
| **Figure S4** | NMDS plots for comparisons on DNA-level |
| **Figure S5** | Correlations between three dominant taxa (control hose) |
| **Figure S6** | Thickness values for real hose biofilm on 2mm-scale |
| **Figure S7** | Correlations between three dominant taxa (real hose) |
| **Figure S8** | TCC for different, theoretically possible sampling sizes |
| **Table S1** | Water characteristics |
| **Table S2** | Settings for Amplification PCR and INDEX PCR reactions |
| **Table S3** | Processing of 16S rRNA gene sequences |
| **Table S4** | Dominant taxa in control hose biofilm |
| **Table S5** | Dominant taxa in real hose biofilm |

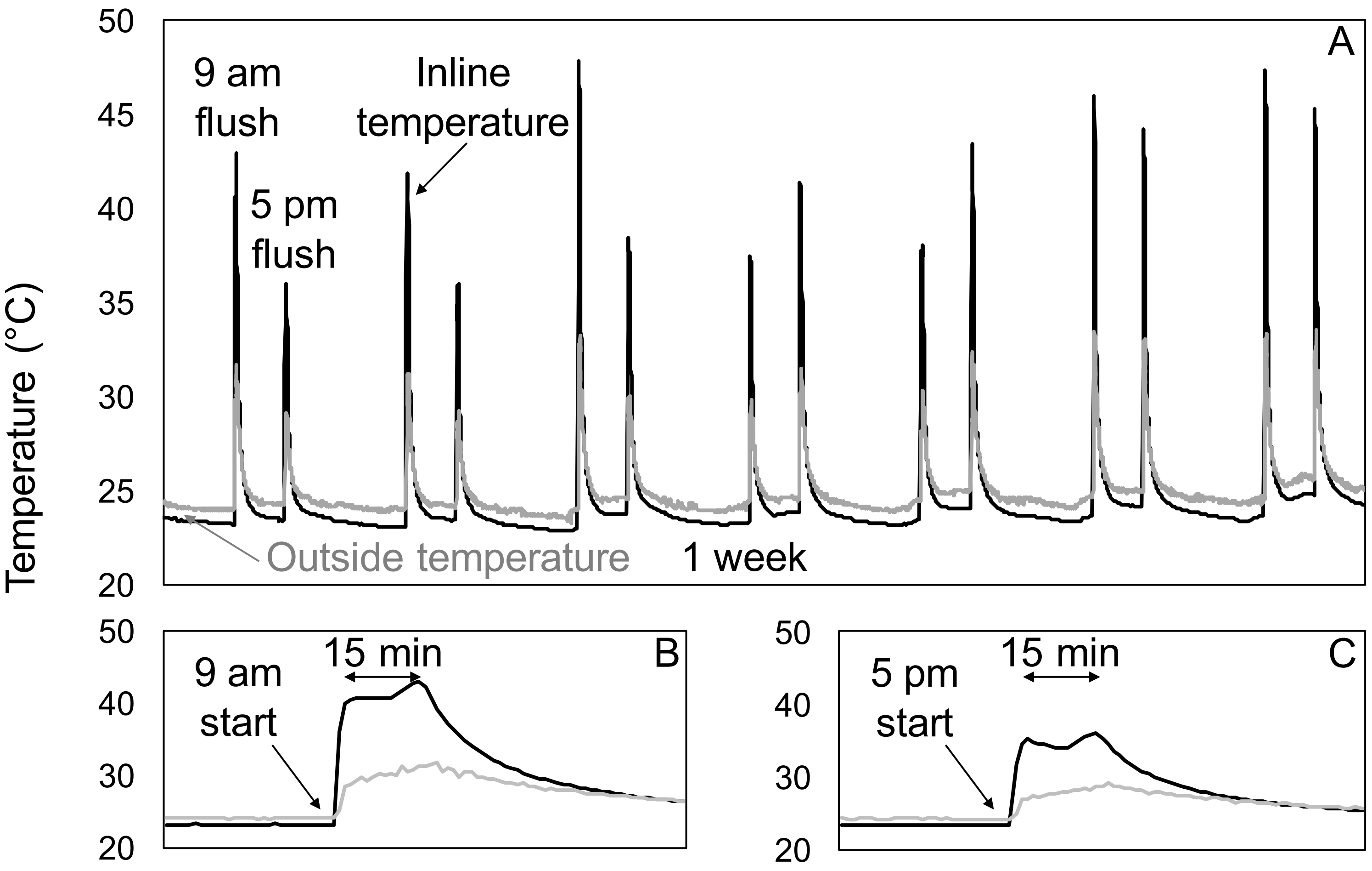


**Supplementary Figure S1.** Temperature pattern inside PVC-P hose (black) and surrounding temperature inside box (gray). (A) over the course of 1 week; in higher resolution, the (B) 9 am flush and (C) 5 pm flush.

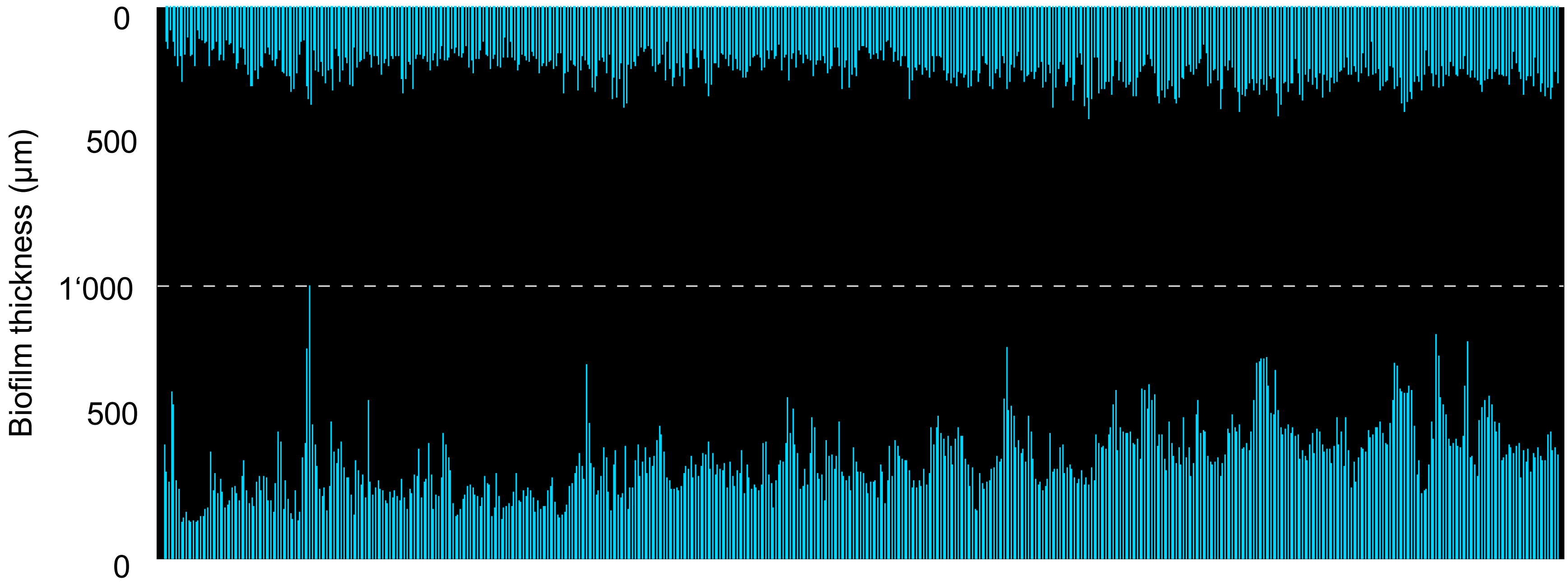


**Supplementary Figure S2.** Thickness of the control hose biofilm. Optical coherence tomography was used for imaging and analyzing structure and thickness of a biofilm grown inside a flexible PVC-P hose under controlled conditions in the laboratory. Images were taken two-dimensional in 2 mm length and 1 mm in height. Here, each bar represents the average thickness for these 2 mm-sections for 1.20 m hose length.

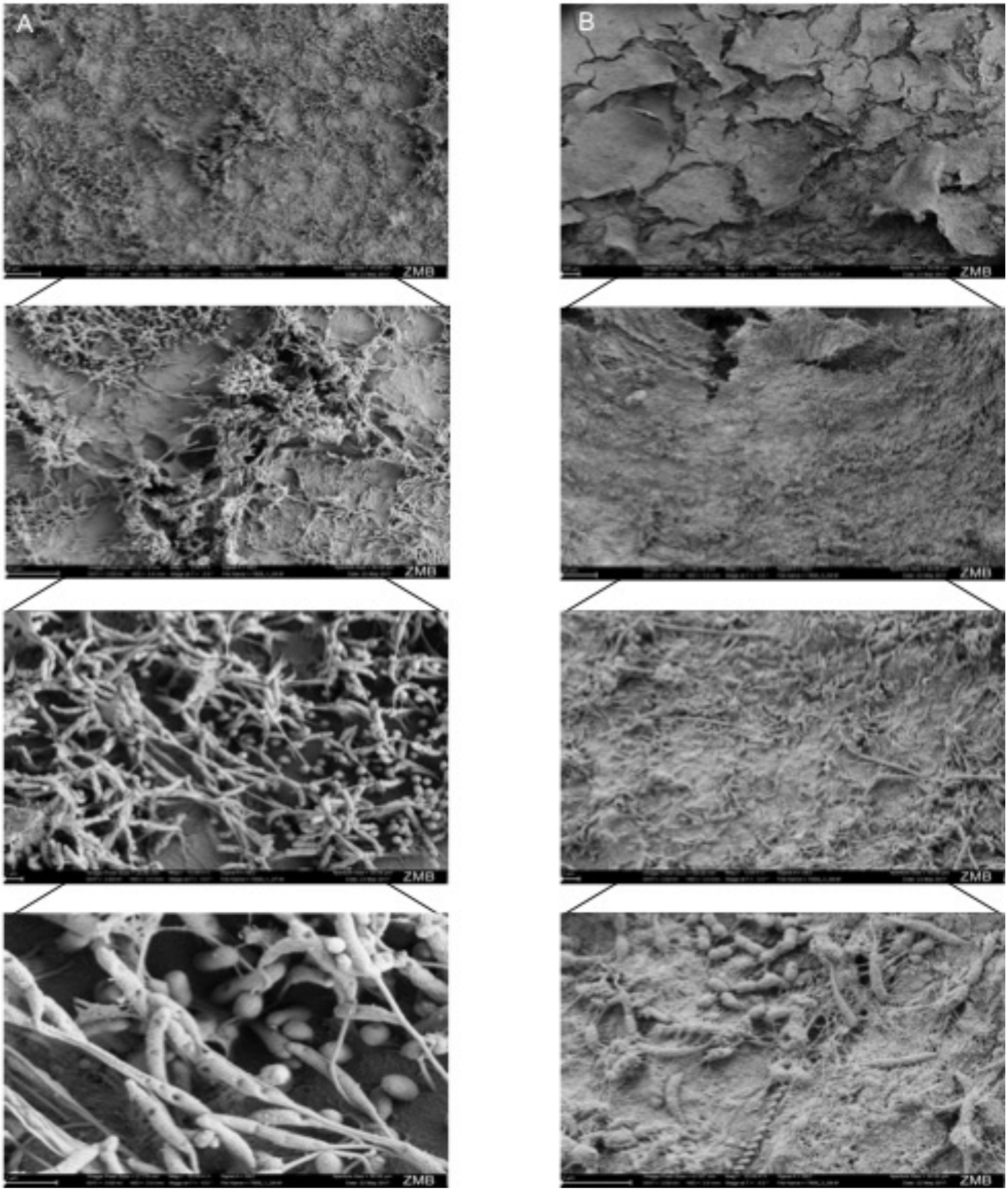


**Supplementary Figure S3.** Scanning electron microscopic image of biofilms grown under (A) controlled laboratory conditions (Control hose) or (B) real use (i.e., uncontrolled) conditions (Real hose). Images made by the Center for Microscopy and Image Analysis, University of Zurich.

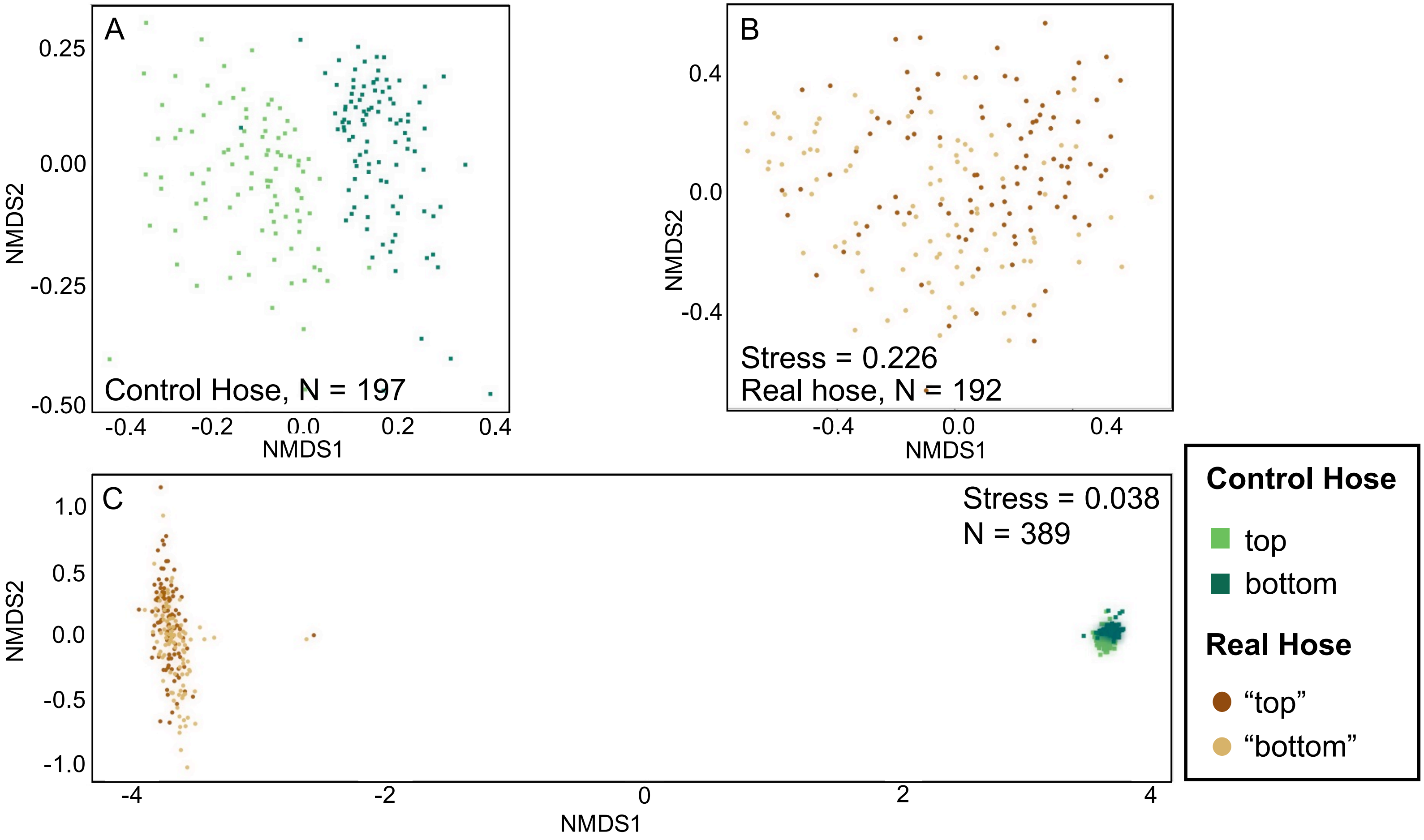


**Supplementary Figure S4.** Non-metric multidimensional scaling representation of bray-curtis dissimilarity between biofilm communities, either grown under controlled laboratory conditions (control hose, A) or under real (i.e., uncontrolled) use conditions (real hose, B). NMDS plots display dissimilarity between subsamples of the control hose biofilm (A), between subsamples of the real hose biofilm (B), and between both biofilms (C).

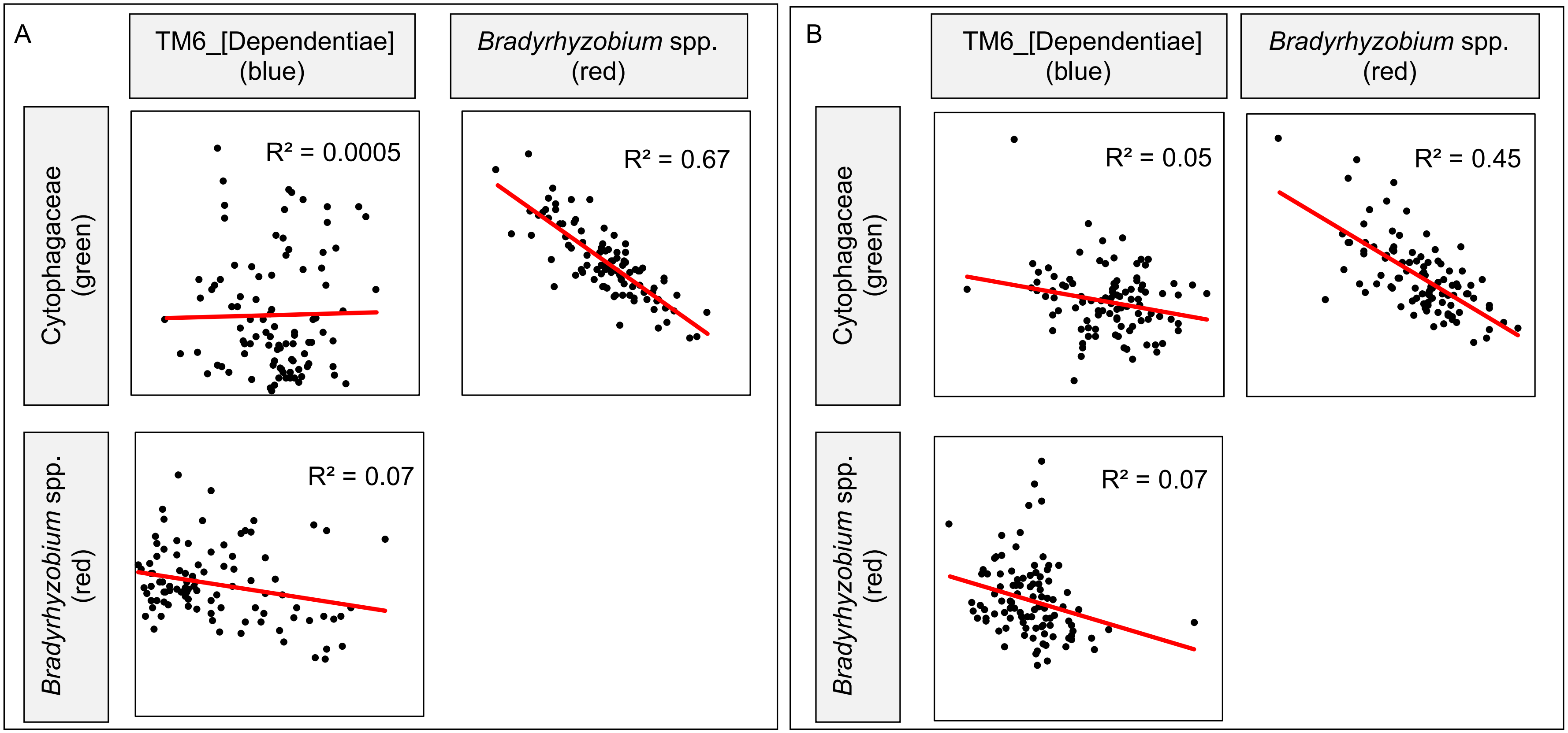


**Supplementary Figure S5.** Correlations between the relative abundances of the three most dominant taxa in the control hose biofilm**.** (A) top, (B) bottom part of the hose. Dominant taxa were identified as *Bradyrhizobium* spp., Cytophagaceae, and TM6_(Dependentiae).

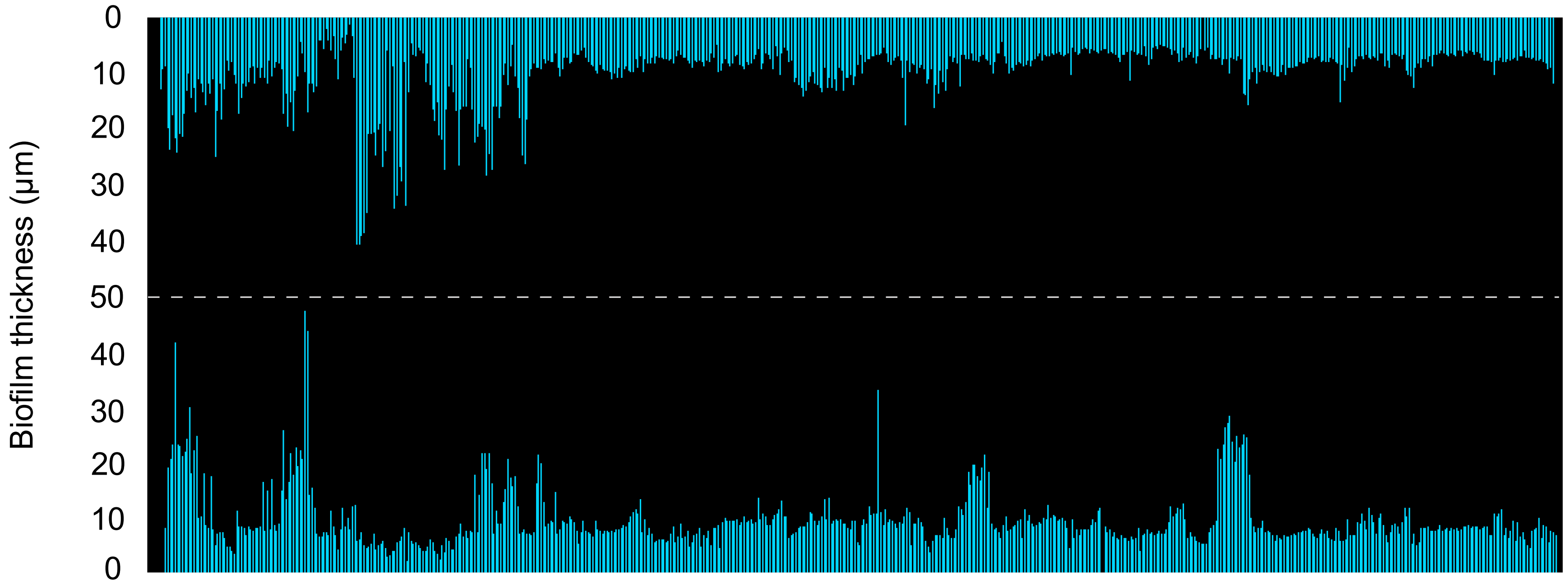


**Supplementary Figure S6.** Thickness of the real hose biofilm. Optical coherence tomography was used for imaging and analyzing structure and thickness of a biofilm grown inside a flexible PVC-P hose under real (i.e., uncontrolled) use conditions. Images were taken two-dimensional in 2 mm length and 1 mm in height. Here, each bar represents the average thickness for these 2 mm-sections for 1.20 m hose length.

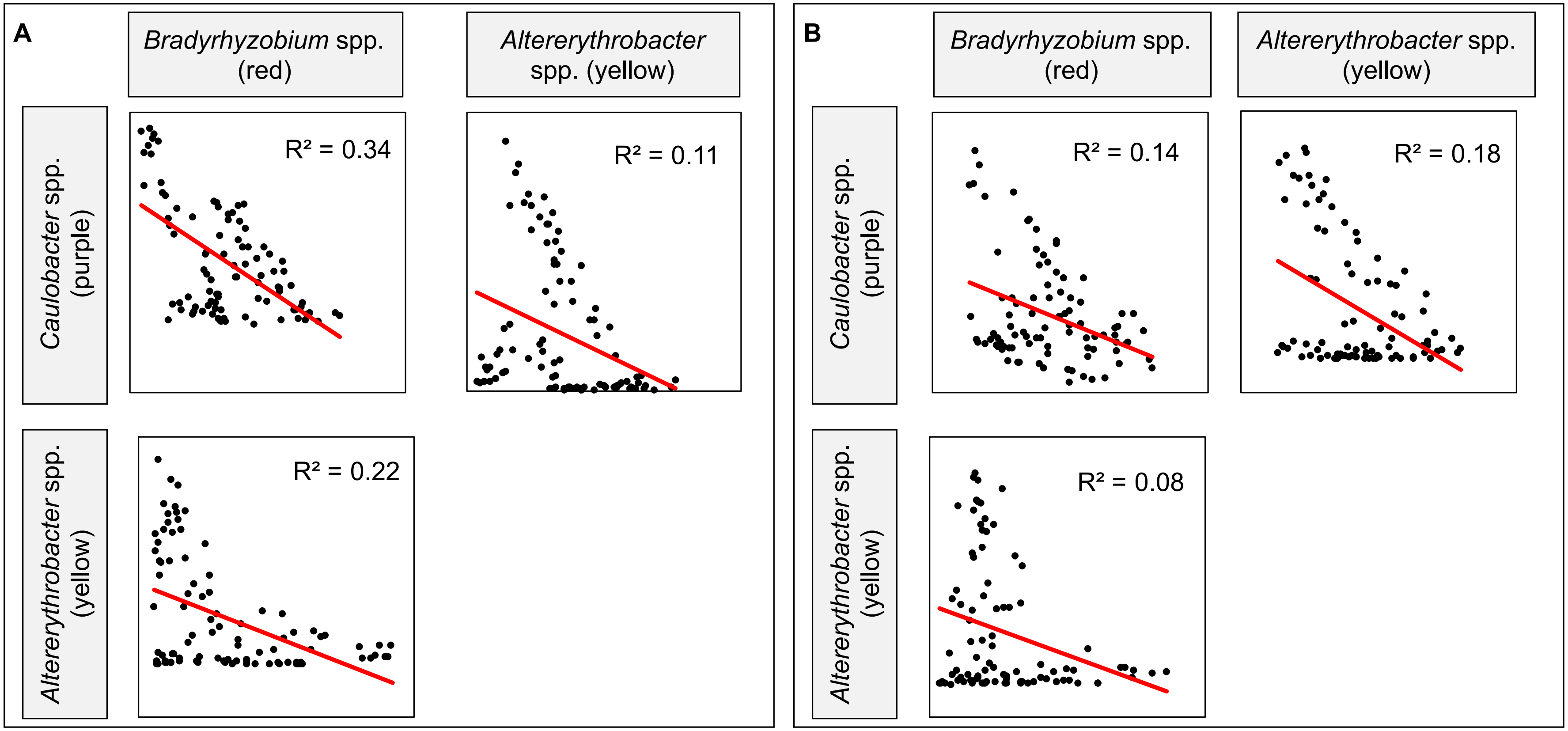


**Supplementary Figure S7.** Correlations between the relative abundances of the three most dominant taxa in the real hose biofilm. (A) top, (B) bottom part of the hose. Dominant taxa were identified as *Bradyrhizobium* spp., *Altererythrobacter* spp., and *Caulobacter* spp.

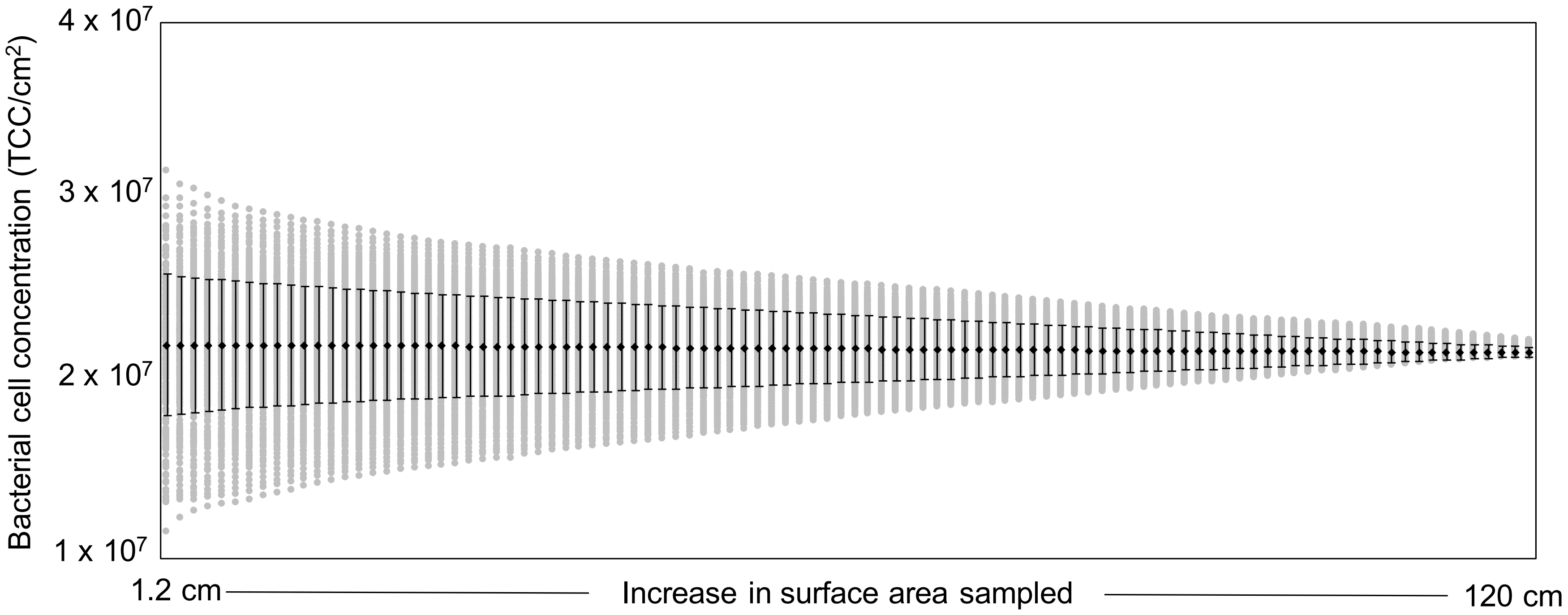


**Supplementary Figure S8.** Relevance of sample size. Bacterial cell numbers of the control hose biofilm were exemplarily used to assess the importance of sample size for the representativeness of results. Samples have been created on 1.2 cm-scale. Here, several sections have been combined to illustrated larger sampling sizes. Black dots represent the overall average for the entire shower hose biofilm. Grey dots illustrate the variation of results, decreasing with increasing sample size (i.e., samples cm in length).

**Supplementary Table S1.** Water characteristics for control and real hose biofilm.

| (A) Control hose | | (B) Real hose |
|---|---|---|
| | **Origin** | |
| 15.0 % | *Ground water* | 96% |
| 6.4 % | *Spring water* | 4% |
| 78.6 % | *Lake water* | - |
| | **Physico-chemical parameters** | |
| 95 mg/L | *Calcium* | 100 mg/L |
| 12 mg/L | *Natrium* | 5 mg/L |
| 14.7 mg/L | *Magnesium* | 15 mg/L |
| 14.3 mg/L | *Nitrate* | 21 mg/L |
| 1.9 mg/L | *Potassium* | - |
| 15.6 mg/L | *Chloride* | 14 mg/L |
| 16.7 mg/L | *Sulfate* | 20 mg/L |
| 0.05 mg/L | *Fluoride* | < 0.1 mg/L |
| - | *Manganese* | > 0.01 mg/L |
| - | *Hydrogen carbonate* | 31 mg/L |
| - | *Iron* | < 0.01 mg/L |
| | | |
| - | *pH* | 7.3 |
| 26.8 °fH | *Hardness* | 34°fH |
| 13.2 °C | *Temperature* | 12°C |
| | | |

**Supplementary Table S2.** Detailed information on PCR reactions

| | | |
|---|---|---|
| **A – Amplicon PCR** | | |
| | | *Volume (25 µL reaction)* |
| 2 x KAPA HiFi HotStart ReadyMix | | 12.5 µL |
| Forward primer (10 µM) | | 0.75 µL |
| Reverse primer (10 µM) | | 0.75 µL |
| Template DNA (adjusted to 1 ng with Dnase free water) | | 11.0 µL |
| | | |
| *Temperature* | *Duration* | *Cycles* |
| 95 °C | 5:00 min | |
| 95 °C | 0:20 min | 29 x |
| 51 °C | 0:15 min | |
| 72 °C | 0:30 min | |
| 4 °C | hold | |
| | | |
| **B – Index PCR** | | |
| | | *Volume (50 µL reaction)* |
| 2 x KAPA HiFi HotStart ReadyMix | | 25.0 µL |
| Nextera XT Index 1 primer | | 5.0 µL |
| Nextera XT Index 2 primer | | 5.0 µL |
| Template DNA | | 15.0 µL |
| | | |
| *Temperature* | *Duration* | *Cycles* |
| 95 °C | 3:00 min | |
| 95 °C | 0:30 min | 8 x |
| 51 °C | 0:35 min | |
| 72 °C | 0:35 min | |
| 4 °C | hold | |

**Supplementary Table S3.** Processing of 16S rRNA gene sequences.

| | | |
|---|---|---|
| **(A)** | **Quality Control** | |
| | **FastQC V0.11.4** | |
| **(B)** | **Trimming and merging of primer sites** | |
| | **usearch** | v10.0.240_i86linux64 |
| | Trim R1 | 20 |
| | Trim R2 | 50 |
| | **Flash** | v1.2.11 |
| | Minimal overlap | 15 |
| | Maximal overlap | 300 |
| | Maximal mismatch density | 0.25 |
| | | |
| | | |
| | | |
| | | |
| **(C)** | **Primer Site Trimming** | |
| | **Usearch v10.0.240 i86linux64** | |
| | Coverage | full-length |
| | Allowed number of mismatches | 1 |
| | Amplicon size range | 50 - 600 |
| | | |
| **(D)** | **Filtering based on quality and size** | |
| | Size range | 200 - 500 |
| | GC range | 30 - 70 |
| | Minimal Q mean | 200 - 500 |
| | Number of Ns | 1 |
| | Low complexity | dust / 30 |

**Supplementary Table S4.** List of all dominant taxa (i.e., with at least 1 % of the total number of reads) in the control hose biofilm.

| Portion of total community (%) | Phylum | Class | Order | Family | Genus |
|---|---|---|---|---|---|
| 24.7 | Bacteroidetes | Cytophagia | Cytophagales | Cytophagaceae | uncultured |
| 23.4 | Proteobacteria | Alphaproteobacteria | Rhizobiales | Bradyrhizobiaceae | Bradyrhizobium |
| 9.6 | TM6_[Dependentiae] | uncultured_bacterium | uncultured_bacterium | uncultured_bacterium | uncultured_bacterium |
| 8.6 | Proteobacteria | Alphaproteobacteria | Rhodobacterales | Rhodobacteraceae | NA |
| 6.3 | Proteobacteria | Alphaproteobacteria | Rhodobacterales | Rhodobacteraceae | Rhodobacter |
| 6.2 | Bacteroidetes | Sphingobacteriia | Sphingobacteriales | Chitinophagaceae | Sediminibacterium |
| 5.7 | Verrucomicrobia | Verrucomicrobiae | Verrucomicrobiales | Verrucomicrobiaceae | Brevifollis |
| 1.9 | Bacteroidetes | Cytophagia | Cytophagales | Cytophagaceae | Ohtaekwangia |
| 1.5 | Proteobacteria | Betaproteobacteria | Rhodocyclales | Rhodocyclaceae | Dechloromonas |
| 1.4 | Proteobacteria | Alphaproteobacteria | Caulobacterales | Caulobacteraceae | Phenylobacterium |
| 1.3 | Proteobacteria | Betaproteobacteria | Rhodocyclales | Rhodocyclaceae | Denitratisoma |

**Supplementary Table S5.** List of all dominant taxa (i.e., with at least 1 % of the total number of reads) in the real hose biofilm.

| Portion of total community (%) | Phylum | Class | Order | Family | Genus |
|---|---|---|---|---|---|
| 34.72 | Proteobacteria | Alphaproteobacteria | Caulobacterales | Caulobacteraceae | Caulobacter |
| 24.24 | Proteobacteria | Alphaproteobacteria | Rhizobiales | Bradyrhizobiaceae | Bradyrhizobium |
| 14.24 | Proteobacteria | Alphaproteobacteria | Sphingomonadales | Erythrobacteraceae | Altererythrobacter |
| 5.41 | Actinobacteria | Actinobacteria | Micrococcales | Brevibacteriaceae | Brevibacterium |
| 4.79 | Proteobacteria | Alphaproteobacteria | Rhizobiales | Bradyrhizobiaceae | Bosea |
| 1.95 | Bacteroidetes | Sphingobacteriia | Sphingobacteriales | Chitinophagaceae | NA |
| 1.36 | Proteobacteria | Deltaproteobacteria | Bdellovibrionales | Bdellovibrionaceae | Bdellovibrio |
| 1.35 | Bacteroidetes | Sphingobacteriia | Sphingobacteriales | Chitinophagaceae | uncultured |
| 1.16 | Proteobacteria | Alphaproteobacteria | Sphingomonadales | Sphingomonadaceae | Sphingomonas |
| 1.15 | Cyanobacteria | ML635J-21 | NA | NA | NA |
| 1.14 | Proteobacteria | Alphaproteobacteria | Rhodobacterales | Rhodobacteraceae | Rhodobacter |
