# Supplementary material for: Small-Scale Heterogeneity in Drinking Water Biofilms
Source: Front Microbiol. 2019 Oct 29;10:2446. doi: 10.3389/fmicb.2019.02446 (PMC6828615; doi:10.3389/fmicb.2019.02446)
Supplement: Supplementary file 2 [file Data_Sheet_2.pdf]

MiSeq AmpSeq *Heterogeneity Study* Workflow in R

- Project: Small-scale heterogeneity in drinking water biofilms
- User(s): Lise Neu (Lisa.Neu@eawag.ch)

- Data type: 16S AmpSeq Illumina MiSeq PE300
- Date : 23.05.2019

Preface

Biofilm heterogeneity has been characterized on various scales for both natural and engineered ecosystems. This heterogeneity has been attributed to spatial differences in environmental factors. Understanding their impact on localized biofilm heterogeneity in building plumbing systems is important for both management and representative sampling strategies. We assessed heterogeneity within the confined engineered ecosystem of a shower hose by high-resolution sampling (200 individual biofilm sections per hose) on varying scales (μm to m), postulating that a biofilm grown on a single material under uniform conditions should be homogeneous in its structure, bacterial numbers, and community composition. A biofilm grown for 12 months under controlled laboratory conditions, showed homogeneity on large-scale. However, small-scale heterogeneity was clearly observed. For example, biofilm thickness of cm-sections varied up to 4-fold (150 – 750 μm), total cell concentrations (TCC) 3-fold (1.1 – 3.4 x 10^7 cells/cm2), and dominant taxa showed fluctuating trends and localized heterogeneity. A biofilm grown under real (i.e., uncontrolled) conditions developed clearly more heterogeneity on both large- and small-scale, with, e.g., impressive spatial fluctuations of the most dominant taxa. Interestingly, both biofilm communities showed comparably low diversities, with < 400 taxa each, and solely 3 taxa accounting for 57 % and 73 % of the respective community. This low diversity was attributed to a strong selective pressure, originating in migrating carbon from the flexible hoses as major carbon source. High-resolution sampling on various scales allowed for a detailed analysis of spatial heterogeneity in drinking water biofilms. This study gives insight into biofilm structure and community composition on cm-to m-scale and is particularly useful for decisions on sampling strategies in biofilm research and monitoring.

Set R Environment

```
## RESET R ENVIRONMENT
rm(list=ls()) # clean/reset environment

## SET WORKING DIRECTORY
setwd("myfolder")
wd <- getwd()
wd
list.files(wd)
```

Libaries and Packages

```
require("phyloseq")
require("ggplot2")
require("vegan")
require("microbiome")
```

Import and subset data

Data is organised in different categories (highlighted in bold font):

The **Experiment** is subdivided in *Control hose* and *Real hose*, both separated according to the **Orientation** of the hoses (*Bottom*, *Top*, *"Bottom"*, *"Top"*). **Kind** corresponds to samples that were amplified in triplicates for amplification/sequencing control, and **Character** serves for distinguishing between *Experimetal samples* and *Control samples* (e.g., Negitve Control for PCR amplification).

```
## LOAD AND IMPORT DATA
treefile <- "p399_Run171106_Run180108_16S_ZOTU_CLU.tre"
otufile <- "p399_Run171106_Run180108_16S_ZOTU_c97_Count_Sintax.txt"
refseqfile <- "p399_Run171106_Run180108_16S_ZOTU_c97.fa"
mapfile <- "p399_run171106_run180108_MapFile1.txt"

d <- import_qiime(otufilename = otufile, mapfilename = mapfile, treefilename = treefile, refseqf
ilename = refseqfile)


## DATA
d

# phyloseq-class experiment-level object
# otu_table()   OTU Table:         [ 545 taxa and 410 samples ]
# sample_data() Sample Data:       [ 410 samples by 11 sample variables ]
# tax_table()   Taxonomy Table:    [ 545 taxa by 7 taxonomic ranks ]
# phy_tree()    Phylogenetic Tree: [ 545 tips and 544 internal nodes ]
# refseq()      DNAStringSet:      [ 545 reference sequences ]


## SUBSET DATA (for "Experiment"; i.e., control and real hose data still combined)
d.e <- subset_samples(d, Character == "e")
d.e

# phyloseq-class experiment-level object
# otu_table()   OTU Table:         [ 545 taxa and 399 samples ]
# sample_data() Sample Data:       [ 399 samples by 11 sample variables ]
# tax_table()   Taxonomy Table:    [ 545 taxa by 7 taxonomic ranks ]
# phy_tree()    Phylogenetic Tree: [ 545 tips and 544 internal nodes ]
# refseq()      DNAStringSet:      [ 545 reference sequences ]
```

Explore data

```
## Summary
summarize_phyloseq(d)
summarize_phyloseq(d.e)

## Number of counts per Taxa
sort(taxa_sums(d))
sort(taxa_sums(d.e))

## Number of counts per Sample
sort(sample_sums(d))
sort(sample_sums(d.e))

## Read-Count Distribution - Careful this are still raw counts!

#pdf("Raw_Counts_per_sample.pdf", paper="a4")

plot(sample_sums(d.e), xaxt = "n", xlab = "Sample", ylab = "Number of Reads", pch = 16, cex=1, c
ol=rgb(0,0,0,alpha=0.5), main="Read-Counts per Sample", ylim=range(0,max(sample_sums(d))*1.1))
```

Processing experimental data for analysis

Remove ZOTUs with zero counts and singletons

Samples with low counts might not represent diversity very well and should therefore be removed.

```
# DETERMINE NUMBER OF ZOTUs WITH <1 AND/OR =1 READS
sum(taxa_sums(d.e) < 1)

# [1] 14

sum(taxa_sums(d.e) ==1)
# [1] 2

# REMOVE ZOTUs WITH <1 AND/OR =1 READS
d.e.noZ <- prune_taxa(taxa_sums(d.e) > 1, d.e)
d.e.noZ

# phyloseq-class experiment-level object
# otu_table()   OTU Table:         [ 529 taxa and 399 samples ]
# sample_data() Sample Data:       [ 399 samples by 11 sample variables ]
# tax_table()   Taxonomy Table:    [ 529 taxa by 7 taxonomic ranks ]
# phy_tree()    Phylogenetic Tree: [ 529 tips and 528 internal nodes ]
# refseq()      DNAStringSet:      [ 529 reference sequences ]
```

Set minimum depth and seed

```
# CHECK MINIMAL DEPTH
min_depth <- min(sample_sums(d.e))
min_depth

# [1] 5648


# CHANGE MINIMAL DEPTH (based on read-count distribution)
min_depth <- c(25000)
min_depth

# [1] 25000


# SET SEED AND RAREFY EVEN DEPTH
set.seed(1)

d.e.rare <- rarefy_even_depth(d.e.noZ, sample.size = min_depth, replace = TRUE, rngseed = 1)

# `set.seed(1)` was used to initialise repeatable random subsampling.
# Please record this for your records so others can reproduce.
# Try `set.seed(1); .Random.seed` for the full vector
# ...
# 10 samples removed because they contained fewer reads than `sample.size`.
# Up to first five removed samples are:

# B005-2B005-3F054F059F080
# ...
# 2OTUs were removed because they are no longer
# present in any sample after random subsampling

# ...

d.e.rare
# phyloseq-class experiment-level object
# otu_table()   OTU Table:         [ 527 taxa and 389 samples ]
# sample_data() Sample Data:       [ 389 samples by 11 sample variables ]
# tax_table()   Taxonomy Table:    [ 527 taxa by 7 taxonomic ranks ]
# phy_tree()    Phylogenetic Tree: [ 527 tips and 526 internal nodes ]
# refseq()      DNAStringSet:      [ 527 reference sequences ]
```

Subset data in Control and Real hose

```
# CONTROL HOSE BIOFILM
d.e.control <- subset_samples(d.e.rare, Experiment == "c")
d.e.control

# phyloseq-class experiment-level object
# otu_table()   OTU Table:         [ 527 taxa and 197 samples ]
# sample_data() Sample Data:       [ 197 samples by 11 sample variables ]
# tax_table()   Taxonomy Table:    [ 527 taxa by 7 taxonomic ranks ]
# phy_tree()    Phylogenetic Tree: [ 527 tips and 526 internal nodes ]
# refseq()      DNAStringSet:      [ 527 reference sequences ]


# REAL HOSE BIOFILM
d.e.real <- subset_samples(d.e.rare, Experiment == "r")
d.e.real

# phyloseq-class experiment-level object
# otu_table()   OTU Table:         [ 527 taxa and 192 samples ]
# sample_data() Sample Data:       [ 192 samples by 11 sample variables ]
# tax_table()   Taxonomy Table:    [ 527 taxa by 7 taxonomic ranks ]
# phy_tree()    Phylogenetic Tree: [ 527 tips and 526 internal nodes ]
# refseq()      DNAStringSet:      [ 527 reference sequences ]
```

Export for final analysis of sequencing data outside the R environment

```
# EXPORT FOR CONTROL HOSE BIOFILM
ot1 <- as(otu_table(d.e.control),'matrix')
tax1 <- as(tax_table(d.e.control), 'matrix')
write.csv(ot1, file = "Lineage_Control_1.csv")
write.csv(tax1, file = "Lineage_Control_2.csv")


# EXPORT FOR REAL HOSE BIOFILM
ot2 <- as(otu_table(d.e.real),'matrix')
tax2 <- as(tax_table(d.e.real), 'matrix')
write.csv(ot2, file = "Lineage_Reall_1.csv")
write.csv(tax2, file = "Lineage_Real_2.csv")
```

> The exported files of each hose biofilm were combined using excel and subsequent community analysis were conducted within the very same.

NMDS - Community analysis based on Bray-Curtis Dissimilariy

```
ord_all <- ordinate(d.e.rare, method = "NMDS", distance = "bray")
#stress 0.03819
p_NMDS_all <- plot_ordination(physeq = d.e.rare, ordination = ord_all, shape = "Experiment", col
or="Orientation")

p_NMDS_all_number <- plot_ordination(physeq = d.e.rare, ordination = ord_all, shape = "Experimen
t", color="NumberCat")
```

```
OneTransp<-rgb(0.65098,0.38039,0.10196,max=1,alpha=0.5)
TwoTransp<-rgb(0.8745,0.76078,0.490196,alpha=0.25)
ThreeTransp<-rgb(0.50196,0.803921,0.44313725,alpha=0.25)
FourTransp<-rgb(0.00392,0.5215686,0.44313725,alpha=0.25)

One<-rgb(0.65098,0.38039,0.10196)
Two<-rgb(0.8745,0.76078,0.490196)
Three<-rgb(0.50196,0.803921,0.44313725)
Four<-rgb(0.00392,0.5215686,0.44313725)

plot(p_NMDS_all + geom_point(aes(color=(Orientation)),alpha=0.01,size=7) + theme_bw()+scale_shap
e_manual(values=c(15,16))+scale_color_manual(values=c(One,Two,Three,Four))+theme(panel.grid.majo
r = element_blank(), panel.grid.minor = element_blank())+coord_fixed(ratio = 1))

plot(p_NMDS_all_number  + geom_point(aes(color=(NumberCat)),alpha=0.01,size=7) + theme_bw() + sc
ale_color_manual(values=c("gray5","gray10","gray15","gray20","gray25","gray30","gray35","gray40"
,"gray45","gray50","gray55"))+scale_shape_manual(values=c(15,16))+theme(panel.grid.major = eleme
nt_blank(), panel.grid.minor = element_blank())+coord_fixed(ratio = 1))

plot(p_NMDS_all_number  + geom_point(aes(color=(NumberCat)),alpha=0.01,size=7) + theme_bw() + sc
ale_shape_manual(values=c(15,16))+theme(panel.grid.major = element_blank(), panel.grid.minor = e
lement_blank())+coord_fixed(ratio = 1))



BC_all<- phyloseq::distance(d.e.rare,method="bray")
SD_all<-data.frame(sample_data(d.e.rare))
adonis(BC_all~Experiment*Orientation,data=SD_all)
#Experiment explained 66% of all variation in communication, while orientation only explained 2%

#Permutation: free
#Number of permutations: 999

#Terms added sequentially (first to last)

#             Df SumsOfSqs MeanSqs F.Model      R2 Pr(>F)
#Experiment    1    52.111  52.111  793.73 0.65885  0.001 ***
#Orientation   2     1.706   0.853   12.99 0.02157  0.001 ***
#Residuals   385    25.277   0.066         0.31958
#Total       388    79.094                 1.00000
#---
#Signif. codes:  0 ‘***’ 0.001 ‘**’ 0.01 ‘*’ 0.05 ‘.’ 0.1 ‘ ’ 1
#Experiment explained 66% of all variation in communication, while orientation only explained 2%
```

```
BC_control<- phyloseq::distance(d.e.control,method="bray")
SD_control<-data.frame(sample_data(d.e.control))
adonis(BC_control~Orientation,data=SD_control)

#Permutation: free
#Number of permutations: 999

#Terms added sequentially (first to last)
```

```
#            Df SumsOfSqs MeanSqs F.Model      R2 Pr(>F)
#Orientation   1    1.3235  1.3235  54.928 0.21978  0.001 ***
#Residuals   195    4.6986  0.0241         0.78022
#Total       196    6.0221                 1.00000
#---
#Signif. codes:  0 ‘***’ 0.001 ‘**’ 0.01 ‘*’ 0.05 ‘.’ 0.1 ‘ ’ 1
#orientation could explain 22% of community variation.

adonis(BC_control~Orientation*NumberCat,data=SD_control)

#Permutation: free
#Number of permutations: 999

#Terms added sequentially (first to last)

                       Df SumsOfSqs MeanSqs F.Model      R2
#Orientation             1    1.3235 1.32353  84.328 0.21978
#NumberCat              10    1.6138 0.16138  10.282 0.26798
#Orientation:NumberCat  10    0.3381 0.03381   2.154 0.05615
#Residuals             175    2.7466 0.01570         0.45609
#Total                 196    6.0221                 1.00000
                      Pr(>F)
#Orientation            0.001 ***
#NumberCat              0.001 ***
#Orientation:NumberCat  0.002 **
#Residuals
#Total
#---
#Signif. codes:  0 ‘***’ 0.001 ‘**’ 0.01 ‘*’ 0.05 ‘.’ 0.1 ‘ ’ 1
```

```
BC_real<- phyloseq::distance(d.e.real,method="bray")
SD_real<-data.frame(sample_data(d.e.real))
adonis(BC_real~Orientation,data=SD_real)

#Permutation: free
#Number of permutations: 999

#Terms added sequentially (first to last)

#            Df SumsOfSqs MeanSqs F.Model      R2 Pr(>F)
#Orientation   1    0.3827 0.38271  3.5336 0.01826  0.007 **
#Residuals   190   20.5779 0.10830         0.98174
#Total       191   20.9607                 1.00000
#---
#Signif. codes:  0 ‘***’ 0.001 ‘**’ 0.01 ‘*’ 0.05 ‘.’ 0.1 ‘ ’ 1

#orientation could only explain 1.8% of variation.



adonis(BC_real~Orientation*NumberCat,data=SD_real)
```

```
#Permutation: free
#Number of permutations: 999

#Terms added sequentially (first to last)

                       Df SumsOfSqs MeanSqs F.Model      R2
#Orientation             1    0.3827 0.38271  8.9089 0.01826
#NumberCat              10   11.8219 1.18219 27.5199 0.56400
#Orientation:NumberCat  10    1.4533 0.14533  3.3831 0.06933
#Residuals             170    7.3028 0.04296         0.34840
#Total                  191   20.9607                 1.00000
                      Pr(>F)
#Orientation            0.001 ***
#NumberCat              0.001 ***
#Orientation:NumberCat  0.001 ***
#Residuals
#Total
#---
#Signif. codes:  0 ‘***’ 0.001 ‘**’ 0.01 ‘*’ 0.05 ‘.’ 0.1 ‘ ’ 1
```

```
ord_real <- ordinate(d.e.real, method = "NMDS", distance = "bray")
# Run 20 stress 0.2258917
# ... New best solution
# ... Procrustes: rmse 0.00165251  max resid 0.02073589
# *** No convergence -- monoMDS stopping criteria:
#     20: stress ratio > sratmax

#p3 <- plot_ordination(physeq = d.e.real, ordination = ord_real, shape = "Experiment", color = "
Orientation")
#p3


p_NMDS_real <- plot_ordination(physeq = d.e.real, ordination = ord_real, color = "Orientation" )

plot(p_NMDS_real  + geom_point(aes(color=(Orientation)),alpha=0.01,size=7) + theme_bw()+scale_sh
ape_manual(values=c(15))+scale_color_manual(values=c(One,Two,Three,Four))+theme(panel.grid.major
 = element_blank(), panel.grid.minor = element_blank())+coord_fixed(ratio = 1))

p_NMDS_real_number <- plot_ordination(physeq = d.e.real, ordination = ord_real, shape = "Orienta
tion", color="NumberCat")

plot(p_NMDS_real_number  + geom_point(aes(color=(NumberCat)),alpha=0.01,size=7) + theme_bw() +sc
ale_shape_manual(values=c(15,16))+theme(panel.grid.major = element_blank(), panel.grid.minor = e
lement_blank())+coord_fixed(ratio = 1))



ord_control <- ordinate(d.e.control, method = "NMDS", distance = "bray")
# Run 20 stress 0.2536307
# *** No convergence -- monoMDS stopping criteria:
#      1: no. of iterations >= maxit
#     19: stress ratio > sratmax
```

```
#p4 <- plot_ordination(physeq = d.e.control, ordination = ord_control, shape = "Experiment", col
or = "Orientation")
#p4



p_NMDS_control <- plot_ordination(physeq = d.e.control, ordination = ord_control, color = "Orien
tation",shape="Experiment" )

plot(p_NMDS_control  + geom_point(pch=15,aes(color=(Orientation)),alpha=0.01,size=7) + theme_bw(
)+scale_shape_manual(values=c(15))+scale_color_manual(values=c(Three,Four))+theme(panel.grid.maj
or = element_blank(), panel.grid.minor = element_blank())+coord_fixed(ratio = 1))

p_NMDS_control_number <- plot_ordination(physeq = d.e.control, ordination = ord_control, shape =
 "Orientation", color="NumberCat")

plot(p_NMDS_control_number  + geom_point(aes(color=(NumberCat)),alpha=0.01,size=7) + theme_bw()
+scale_shape_manual(values=c(15,16))+theme(panel.grid.major = element_blank(), panel.grid.minor
= element_blank())+coord_fixed(ratio = 1))
```

NEGATIVE AND POSITIVE CONTROLS

Subset data for analysing control samples

```
d.n <- subset_samples(d, Character == "d")
d.n

# phyloseq-class experiment-level object
# otu_table()   OTU Table:         [ 545 taxa and 11 samples ]
# sample_data() Sample Data:       [ 11 samples by 11 sample variables ]
# tax_table()   Taxonomy Table:    [ 545 taxa by 7 taxonomic ranks ]
# phy_tree()    Phylogenetic Tree: [ 545 tips and 544 internal nodes ]
# refseq()      DNAStringSet:      [ 545 reference sequences ]
```

> 11 samples: 9 negative and 2 positive controls.

Negativ controls

There were two kinds of negative controls (NCs):

(1) Controls taken to evaluate the potential of cross contamination during & between experimental processes, namely biofilm removal and needle sonication. These NCs are not necessarily expected to show zero or low counts.

(2) Controls taken for potential contamination steps during the experiment, namely filtration for DNA analyis, process of DNA extraction, and process of PCR amplification. All of these samples are expected to show zero or low counts.

```
Negative sample description:
# NC-1-17    Control hose, Sample processing: Biofilm removal (brushing 10 mL of filtered water in petri dish), followed by needle sonication
             -> expectation = a decent number of counts, but acceptable for further analysis
# NC-1-18    Real hose, Sample processing: Biofilm removal, followed by needle sonication
             -> expectation = decent number of counts, but acceptable for further analysis
# NC-2       Real hose: Ultrapure water on sterile 0.2 µm filter
             -> expectation = zero (or low) counts
# NC-3       Control hose, Sample processing: Biofilm removal, followed by needle sonication
             -> expectation = decent number of counts, but acceptable for further analysis
# NC-4       Control hose: 0.2 µm filtered bottled Evian (used as matrix for
             biofilm suspension)
             -> expectation = zero (or low) counts
# NC-DNA     Real hose: sterile water extracted
             -> expectation = zero (or low) counts
# NC-Ext     Control hose: sterile water extracted
             -> expectation = zero (or low) counts (Real hose)
# NC-PCR-17  Control hose, PCR blanks -> expectation = zero (or low) counts
# NC-PCR-18  Real hose, PCR blanks -> expectation = zero (or low) counts
```

Negative control raw data was processed in the very same way as the real biofilm data. I.e., removing taxa with less than 2 reads and rarefication to an even sampling depth of 25'000 reads/sample. Due to these restrictions, only 2 Negative controls showed positive reads for some taxa, the remaining controls were excluded during this process due to low total number of reads. By this, all Negative controls of the Real hose experiment, all DNA extraction controls, and PCR negative controls were excluded.

For the remaining Negative controls, the presence and relative abundance of the identified taxa (with relative abundance > 1% of the total number of reads) was compared to taxa identified in the corresponding data set (i.e, control hose biofilm data).

ZOTU	Identiy (Genus level)	Negative control "NC-1-17"(rel%)	Negative control "NC-3" (rel%)	Control hose biofilm (averaged rel%)
3	uncultured (Family: Cytophagaceae)	39.7	8.0	24.7
15	Bradyrhizobium	13.4	3.4	23.4
6	NA (Family: Comamonadaceae)	10.8	3.2	8.6
5	uncultured (Phylum: TM6)	10.4	5.9	9.6
7	NA (Family: Rhodobacteraceae)	3.3	2.2	6.3
814	NA (Family: P3OB-42)	2.6	-	0.5
63	Turneriella	2.2	-	0.4
187	Denitratisoma	1.8	1.5	5.7
466	Acinetobacter	1.5	-	0.0
14	Sediminibacterium	1.2	-	1.9
259	Acinetobacter	1.0	-	0.0
56	Pseudorhodoferax	-	67.1	0.0
714	Variovorax	-	1.4	0.0

> The majority of taxa identified in the Negative controls ("NC-1-17", "NC-3") were not dominant in the data of the control hose biofilm. However, for "NC-1-17", ZOTU3 and ZOTU15 showed a relative abundance of 39.7% and 13.4% respectively. Both these taxa were also amongst the most dominant ones in the Control hose biofilm community. In "NC-3", ZOTU3 showed a relaitve abundance of 7.7% and ZOTU5 one of 5.5%. As both these Negative controls were *processing controls* and the observed ZOTUs not present in the other tested negative controls (checked even though number of reads below 25'000 and thus excluded) it is supposedly a result of biofilm/DNA transfer between individual samples. It is therefore suggested to clean toothbrush heads in a way that also removes remainind DNA, e.g., with a chlorination step. For this study, transfer of biofilm material was minimized as good as possible (in the skope of the experiment). It is presumed, that the identified ZOTUs were dominant thoughout the biofilm(s), as the contamination level would otherwise have been diluted out.Nevertheless, the high number of reads (i.e., relative abundance) for ZOTU3 in the Processing Negative controls ("NC-1-17" and "NC-3") cannot entirely be neglected and should therefore been taken into account when drawing conclusions on small-scale heterogeneity for this taxon.

Positiv controls

As a control for the PCR reaction and MiSeq sequencing, a positive control was amplified in addition to the actual samples (for both control and real hose separately). Here, a selfmade MOCK community (by the Genetic Diversity Center, ETH Zürich) was used, consisting of: *Burkholderia xenovorans*, *Bacillus subtilus*, *Escherichia coli*, *Micrococcus luteus*, *Pseudomonas protegens*, *Paenibacillus sabinae*, and *Streptomycetes violaceoruber*. The success of their amplification was verified, following the same data processing steps as for the Negative controls (see above).

The presence and abundance of each Positive control taxon was checked and compared between the sequencing run of both Control and Real hose.

ZOTU	Identiy (Genus level)	Control hose biofilm (rel%)	Real hose biofilm (rel%)
65	Burholderia-Paraburkholderia	41.62	34.19
68	Bacillus	49.83	55.24
122	Escherichia-Shigella	4.04	3.72
121	Micrococcus	0.02	0.03
347	Pseudomonas	0.36	0.33
808	Paenibacillus	0.13	0.14
499	Streptomyces	0.06	0.03

> All taxa that were supposed to be identified within the amplified and sequenced MOCK community were detectable (not on species but genus level). As the positive control was run for both control and real hose biofilms, the amplification success could be compared between the two sequencing runs.
>
> > In the table above, the relative percentages of the members of the MOCK community are displayed (rarefied to an even depth of 25'000 reads/sample to allow for comparability to the main experiemental samples). As the relative % of the genera is comparable between the sequencing of control and real hose biofilms, the amplification of the Positive control can be evaluated as successful.

Replicate sequencing of specifc samples

For the evaluation of the sequencing data, some samples have been amplified and sequenced in replicates. Here, 1 sample of the control hose was amplified in triplicate ("U093") as well as 6 samples of the real hose ("B005", "B013", "B027", "B042", "B050", "B082").

Subset data set

```
# REPLICATE SAMPLES FOR AMPLIFICATION CONTROL
d.t <- subset_samples(d, Kind == "t")
d.t

# phyloseq-class experiment-level object
# otu_table()   OTU Table:         [ 545 taxa and 20 samples ]
# sample_data() Sample Data:       [ 20 samples by 11 sample variables ]
# tax_table()   Taxonomy Table:    [ 545 taxa by 7 taxonomic ranks ]
# phy_tree()    Phylogenetic Tree: [ 545 tips and 544 internal nodes ]
# refseq()      DNAStringSet:      [ 545 reference sequences ]
```

Remove ZOTUs with zero counts and singletons

Samples with low counts might not represent diversity very well and should therefore be removed.

```
# DETERMINE NUMBER OF ZOTUs WITH <1 AND/OR =1 READS
sum(taxa_sums(d.t) < 1)
[1] 252

sum(taxa_sums(d.t) == 1)
[1] 25

d.t.noZ <- prune_taxa(taxa_sums(d.t) > 1, d.t)
d.t.noZ

# phyloseq-class experiment-level object
# otu_table()   OTU Table:         [ 268 taxa and 20 samples ]
# sample_data() Sample Data:       [ 20 samples by 11 sample variables ]
# tax_table()   Taxonomy Table:    [ 268 taxa by 7 taxonomic ranks ]
# phy_tree()    Phylogenetic Tree: [ 268 tips and 267 internal nodes ]
# refseq()      DNAStringSet:      [ 268 reference sequences ]
```

Set minimum depth and seed

```
# CHECK MINIMAL DEPTH
min_depth <- min(sample_sums(d.e))
min_depth

# [1] 5648


# CHANGE MINIMAL DEPTH (based on read-count distribution)
min_depth <- c(25000)
min_depth

# [1] 25000


# SET SEED AND RAREFY EVEN DEPTH
set.seed(1)

d.t.rare <- rarefy_even_depth(d.t.noZ, sample.size = min_depth, replace = TRUE, rngseed = 1)

# `set.seed(1)` was used to initialize repeatable random subsampling.
# Please record this for your records so others can reproduce.
# Try `set.seed(1); .Random.seed` for the full vector
# ...
# 2 samples removed because they contained fewer reads than `sample.size`.
# Up to first five removed samples are:

# B005-2B005-3
# ...
# 24OTUs were removed because they are no longer
# present in any sample after random subsampling


d.t.rare
# phyloseq-class experiment-level object
# otu_table()   OTU Table:         [ 244 taxa and 18 samples ]
# sample_data() Sample Data:       [ 18 samples by 11 sample variables ]
# tax_table()   Taxonomy Table:    [ 244 taxa by 7 taxonomic ranks ]
# phy_tree()    Phylogenetic Tree: [ 244 tips and 243 internal nodes ]
# refseq()      DNAStringSet:      [ 244 reference sequences ]
```

Export for final analysis of sequencing data outside the R environment

```
# EXPORT FOR ALL REPLICATE SAMPLES
ot3 <- as(otu_table(d.t.rare),'matrix')
tax3 <- as(tax_table(d.t.rare), 'matrix')
write.csv(ot3, file = "Lineage_Replicates_1.csv")
write.csv(tax3, file = "Lineage_Replicates_2.csv")
```

> The exported files of each hose biofilm were combined using excel and subsequent community analysis were conducted with the very same.

Sample "B005" could not be used for replicate analysis as 2 out of 3 samples were removed during rarefication. Also, "B-027" was removed from further analysis as only 2 replicates remained.

10 most dominant ZOTUs	average (B013)	stdev (+/-)	average (B042)	stdev (+/-)	average (B050)	stdev (+/-)	average (U082)	stdev (+/-)	average (U093)	stdev (+/-)
#1	15'338	200	10'005	253	14'288	344	8'254	22	8'946	250
#2	4'436	133	6'821	59	3'565	64	6'338	12	3'883	116
#3	2'051	63	1'594	99	2'124	117	6'234	91	3'001	94
#4	1'394	25	1'354	50	1'413	73	1'172	17	2'178	68
#5	309	37	1'033	29	579	19	948	80	1'406	39
#6	288	23	613	22	526	21	260	22	1'404	76
#7	229	25	547	27	338	38	245	8	1'214	27
#8	130	5	422	18	290	18	228	20	427	16
#9	119	7	413	41	258	29	103	10	401	43
#10	81	12	245	22	233	14	101	20	204	6

> Percentages of standard deviations in the total number of reads varied between 0.2 - 19.7 % (average: 5.4 ± 4.0%, n = 50), considering the ten most dominant ZOTUs of individual (triplicate) samples.
